# Cell‐Inspired All‐Aqueous Microfluidics: From Intracellular Liquid–Liquid Phase Separation toward Advanced Biomaterials

**DOI:** 10.1002/advs.201903359

**Published:** 2020-02-11

**Authors:** Qingming Ma, Yang Song, Wentao Sun, Jie Cao, Hao Yuan, Xinyu Wang, Yong Sun, Ho Cheung Shum

**Affiliations:** ^1^ Department of Pharmaceutics School of Pharmacy Qingdao University Qingdao 266021 China; ^2^ Wallace H Coulter Department of Biomedical Engineering Georgia Institute of Technology & Emory School of Medicine Atlanta GA 30332 USA; ^3^ Center for Basic Medical Research TEDA International Cardiovascular Hospital Chinese Academy of Medical Sciences & Peking Union Medical College Tianjin 300457 China; ^4^ Institute of Applied Mechanics National Taiwan University Taipei 10617 Taiwan; ^5^ Institute of Thermal Science and Technology Shandong University Jinan 250061 China; ^6^ Department of Mechanical Engineering University of Hong Kong Pokfulam Road Hong Kong; ^7^ HKU‐Shenzhen Institute of Research and Innovation (HKU‐SIRI) Shenzhen 518000 China

**Keywords:** advanced biomaterials, all‐aqueous microfluidics, cell‐inspiration, intracellular organelles, liquid–liquid phase separation

## Abstract

Living cells have evolved over billions of years to develop structural and functional complexity with numerous intracellular compartments that are formed due to liquid–liquid phase separation (LLPS). Discovery of the amazing and vital roles of cells in life has sparked tremendous efforts to investigate and replicate the intracellular LLPS. Among them, all‐aqueous emulsions are a minimalistic liquid model that recapitulates the structural and functional features of membraneless organelles and protocells. Here, an emerging all‐aqueous microfluidic technology derived from micrometer‐scaled manipulation of LLPS is presented; the technology enables the state‐of‐art design of advanced biomaterials with exquisite structural proficiency and diversified biological functions. Moreover, a variety of emerging biomedical applications, including encapsulation and delivery of bioactive gradients, fabrication of artificial membraneless organelles, as well as printing and assembly of predesigned cell patterns and living tissues, are inspired by their cellular counterparts. Finally, the challenges and perspectives for further advancing the cell‐inspired all‐aqueous microfluidics toward a more powerful and versatile platform are discussed, particularly regarding new opportunities in multidisciplinary fundamental research and biomedical applications.

## Graphical abstract

Inspired by the liquid–liquid phase separation of membraneless organelles, all‐aqueous microfluidics exploit an aqueous two‐phase system to tailor the aqueous structures and shape the formation of materials. This approach is biocompatible for encapsulation, assembly, and patterning of biomolecules and cells in a near‐physiological environment, which enables the state‐of‐art design of advanced biomaterials with exquisite structural proficiency and diversified biological functions.

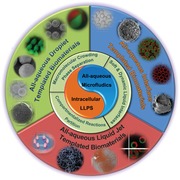

## 1 Introduction

Nature has evolved for billions of years to develop elaborate and sophisticated living organisms,^1^, ^2^, ^3^, ^4^, ^5^, ^6^, ^7^, ^8^, ^9^ which can provide sources of inspired solutions for various intriguing and enduring scientific and technique questions.^10^, ^11^, ^12^, ^13^ Although a single cell is the simplest unit of living organisms, it is rich in high degrees of structural and functional complexities.^14^, ^15^, ^16^, ^17^ The amazing and vital role of cells in life have attracted great scientific interests for constructing “artificial cells” by combining biological or nonbiological components to form microscaled assemblies that are intended to mimic one or more aspects of cells,^18^, ^19^, ^20^, ^21^, ^22^ such as the semipermeable membrane assembly and the ability to perform a simple reaction in their interiors.^23^, ^24^, ^25^, ^26^ The development of artificial cellular systems has led to progresses in understanding the origin of life, connecting the nonliving to the living world, cell biology, and other areas such as biotechnology and biomedical applications where cell‐inspirations can be adopted in new ways.^21^, ^27^, ^28^, ^29^, ^30^, ^31^, ^32^

While synthetic biology and artificially cellular systems are being developed, the current mimicry is mainly stayed at an infant stage and far from completion.^33^, ^34^, ^35^, ^36^ It is important to note that the interior of cells at subcellular level is also very essential and is a treasury with inspirations for further investigation.^37^, ^38^, ^39^, ^40^ Specifically, the biological intracellular environment is heterogeneous and full of distinct compartments with different compositions and properties.^40^, ^41^ For example, membrane‐bound organelles such as the nucleus and mitochondria are surrounded by proteolipid membranes, while membraneless organelles are dynamic and stoichiometric stable complexes, mainly identified as RNA/protein coacervates in nucleolus.^42^, ^43^, ^44^, ^45^, ^46^ Many key intracellular processes, including biomolecular localization, signaling, structuring of cytoplasm, as well as the protection of active components in cells rely on these organelles.^47^, ^48^, ^49^, ^50^, ^51^, ^52^, ^53^, ^54^ These intracellular structures have sparked growing research interests not only for their significant performances, but more importantly, for the scenario that they are assembled and compartmentalized through liquid–liquid phase separation (LLPS) which occurred spontaneously at mild conditions.^55^, ^56^, ^57^ All of these discoveries about the intracellular structures can be used as important guidelines and design principles for developing cell‐inspired biomaterials, which would show great potentials in a broad range of biomedical applications, such as delivery of drugs and vaccine for therapeutic purposes,^58^, ^59^ smart sensors for biomedical imaging and disease diagnosis,^60^, ^61^ soft robotics with stimuli‐responsive and environmentally adaptive performances,^62^, ^63^ as well as force‐bearing and wound healing scaffolds for tissue engineering.^64^, ^65^

Various templates, such as solid particles,^66^ air bubbles,^67^ and liquid structures,^68^ have been introduced for the fabrication of these biomaterials with tunable permeability,^69^ defined geometry,^70^ and desired functionalities.^71^ Among them, all‐aqueous emulsions generated from physical LLPS represent the most biocompatible option^72^ for the assembly of biomolecules and synthesis of biomaterials in a physiological‐like environment. LLPS is a recently appreciated biological route to spontaneously form intracellular structures in living cells,^25^, ^73^ as well as a common physical phenomenon in aqueous polymer solutions;^74^, ^75^ therefore, it is relatively straightforward to utilize LLPS as the way to create suitable templates to mimic the intracellular environment and assemble the cell‐inspired biomaterials with minimized deleterious effects of the active ingredients.^76^ Moreover, precise control over the all‐aqueous structures can be achieved by microfluidic technology, which further facilitates the fabrication of desired biomaterials, developed in the last decade.^77^, ^78^, ^79^, ^80^, ^81^, ^82^ Here, we summarize up‐to‐date progress in all‐aqueous microfluidics, from fluidic fundamentals mimicking the intracellular organelles to fabrication of cell‐inspired advanced biomaterials.

**Figure 1:**
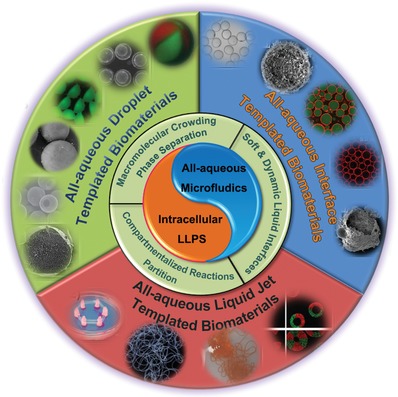
An overview illustrating the key points of cell‐inspired all‐aqueous microfluidics, including the mimicry of intracellular LLPS of all‐aqueous emulsions and the assembly of cell‐inspired biomaterials for emerging biomedical applications.

Structure of this review (**Figure** **1**) follows the basic principles of developing bioinspired materials:^59^, ^83^ 1) the elucidation of the characteristics of all‐aqueous emulsions for mimicking the intracellular LLPS in living cells; 2) the elaborate control over the all‐aqueous emulsions by microfluidics as biocompatible templates for subsequent design and fabrication; 3) integration of designed structure with functions to fulfill specific applications. Emerging applications benefits from the all‐aqueous microfluidics are reviewed, highlighting the beauty of fundamental science and envision of biomedical applications. The challenges and perspectives on developing new all‐aqueous microfluidics and novel biomaterials with innovative applications are proposed and discussed in the end.

## 2 All‐Aqueous Emulsions: Learning from Intracellular LLPS

Recently, intracellular LLPS has been recognized as the possible answer to the enduring biological question of how the incredible structural and functional complexity of modern cells are evolved from a simple and primordial environment contains different macromolecules with presumably dilute concentrations.^84^, ^85^, ^86^ Specifically, the thermodynamic incompatibility of macromolecules leads to intracellular LLPS, which subsequently triggers the “macromolecular crowding”^87^ of the intracellular environment, resulting in different phase‐separated microcompartments. These intracellular microcompartments have soft and dynamic interfaces, by which the encapsulated biomolecular components are able to exchange with the surrounding cytoplasm or nucleoplasm based on their preferential partitioning properties.^25^ Consequently, different biomolecules are enriched in particular microcompartments and facilitates the compartmentalized reactions for the synthesis of proteins and nucleic acids.^88^, ^89^, ^90^ Therefore, when looking for inspirations from intracellular environment for developing biomaterials, the selected templates should be able to mimic the primary characteristics of the intracellular LLPS.

All‐aqueous emulsions, also called aqueous two‐phase system (ATPS), are immiscible aqueous phases generated by physical LLPS in aqueous polymer solutions.^77^, ^91^, ^92^, ^93^, ^94^ This emulsion system consists of droplets of one aqueous phase dispersed into the other aqueous phase. To maintain the immiscibility, the aqueous mixtures are dissolved with at least two solute species, and each solute species are typically enriched in one aqueous phase.^95^ The thermodynamic incompatibility between certain combinations of hydrophilic molecules is the key to trigger its construction.^96^ In this section, we review the distinct characteristics of all‐aqueous emulsions with a focus on their similarities with intracellular LLPS to demonstrate their superior feasibility as templates for developing cell‐inspired biomaterials.

### 2.1 Macromolecular Crowding and Phase Separation

**Figure 2:**
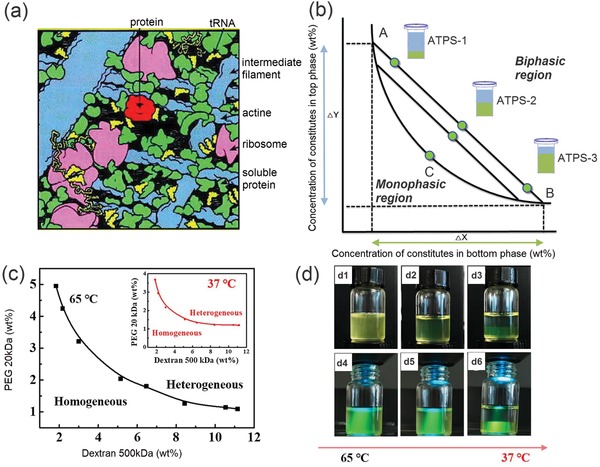
Intracellular macromolecular crowding and phase separation of ATPS. a) Schematics of the macromolecular crowding in eukaryotic cytoplasm. b) Schematics of the phase diagram of a typical aqueous two‐phase system. c) Phase diagrams of PEG/dextran ATPS at 65 and 37 °C (inset). d) Phase separation of the PEG/dextran ATPS when cooling down from 65 to 37 °C. The brightfield (top) and corresponding fluorescence photos of the ATPS are taken (bottom). a) Reproduced with permission.^87^ Copyright 2001, American Society for Biochemistry and Molecular BI. c,d) Reproduced with permission.^113^ Copyright 2018, Elsevier.

A characteristic of the interiors of cells is the high total amount of macromolecules they contain. The interiors are “crowded” rather than “concentrated” since no single macromolecule is at high concentration;^97^, ^98^ but when taken together, they occupy a significant high space fraction (typically 20–30%) of the total interior volume.^74^ This sterically excluded volume effect, also termed “macromolecular crowding,” has considerable impacts on a wide range of intracellular structures and phenomenon;^87^, ^99^, ^100^ for instance, it can lead to the oligomerization of the macromolecules which would then trigger the aggregation and depletion effect of the oligomeric biomolecules, and subsequently results in LLPS^101^, ^102^, ^103^ to form the intracellular microcompartments.^104^, ^105^, ^106^ **Figure 2**a provides a schematic illustration of the macromolecular crowding in eukaryotic cytoplasm.^87^

Such an important phenomenon can be studied and mimicked through all‐aqueous emulsions. Typically, in the classical example of an ATPS, the polymers used are dextran and poly(ethylene glycol) (PEG).^107^, ^108^ When increasing the concentrations, the two polymers will first self‐aggregate and gradually phase separates with each other to change the homogeneous solution into a biphasic system, as illustrated in the phase diagram of Figure 2b. This process is similar to that the cell interiors undergo macromolecular crowding and phase separate to form intracellular microcompartments. Moreover, similar to the intracellular LLPS, ATPS is also a dynamic entity sensitive to environmental changes, such as fluctuations in pH, temperatures, and local concentrations of other biomolecules.^109^, ^110^, ^111^ For example, the phase separation of dextran/PEG is affected by temperature variations; when a homogeneous phase with its composition slightly below the binodal curve at 65 °C, it will separate into heterogeneous phases upon the temperature drops to 37 °C, as shown by the phase diagram in Figure 2c and the digital photos in Figure 2d.^112^, ^113^

### 2.2 Soft and Dynamic Liquid Interfaces

After formation of intracellular microcompartments induced by macromolecular crowding, the maintenance of their morphology relies on the intracellular interfacial tensions.^114^, ^115^ The intracellular structures, like the membraneless organelles, hold very soft liquid interfaces with ultralow interfacial tensions reported to be in the ≈1–10 µN m^−1^ range.^116^, ^117^, ^118^ The ultralow interfacial tensions not only act as the driving force to hold the intracellular morphology, but also contribute to the dynamic performance of the intracellular structures.^119^ Specifically, the intracellular components are able to exchange with the surrounding environment through the liquid–liquid interfaces;^120^, ^121^ therefore, individual microcompartment has the ability to evolve into more sophisticated structures with different subcompartments. For example, the dynamic exchange of proteins and RNA between the nucleolar microcompartments and the nucleoplasm will induce the variations in interfacial tensions of the nucleoli and finally turn it into coexisting and immiscible nucleolar subcompartments with core–shell morphology.^118^

**Figure 3:**
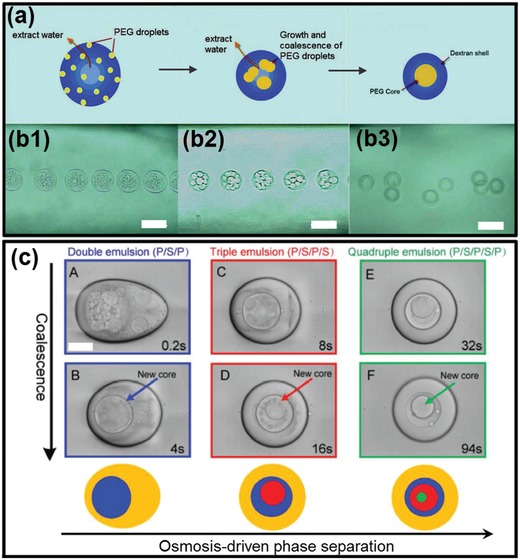
Mimicry of sophisticated intracellular LLPS dynamically changes the all‐aqueous interfaces. a,b) Formation of all‐aqueous emulsions with triple compartments induced by phase separation inside single ATPS; scale bars: 100 µm. c) Time series of optical microscope images showing the osmosis‐induced changing of all‐aqueous emulsions from initial single‐phase into heterogeneous level with multicompartments; scale bar: 200 µm. a,b) Reproduced with permission.^127^ Copyright 2012, American Chemical Society. c) Reproduced with permission.^126^ Copyright 2018, Wiley‐VCH.

All‐aqueous emulsions perform parallel interfacial properties compared with intracellular LLPS. Particularly, the interfacial tensions between two immiscible aqueous phases are also ultralow, often in the range of ≈5 × 10^−4^–0.5 mN m^−1^ which is comparable to that of intracellular interfacial tensions.^122^, ^123^, ^124^ For instance, the typical interfacial tensions for dextran/PEG ATPS is only 0.07 mN m^−1^ when the concentration of dextran and PEG is 8 and 6 wt%, respectively, which is around four hundred times smaller than that of oil–water interfaces (in the range of 30 mN m^−1^).^95^, ^96^, ^125^ The ultralow interfacial tension turns the all‐aqueous emulsions into a physically active system that is extremely sensitive to external influences. Specifically, the pressure between the two aqueous phases may not provide enough shear force to cause the droplets to pinch. The all‐aqueous interfaces are inclined to deform even subjected to tiny external vibrations, therefore the creation of new liquid–liquid interfaces only consumes very little energy.^78^, ^79^, ^80^ Moreover, the aqueous–aqueous interfaces are also highly dynamic, making them versatile templates for developing different biomaterials to replicate the intracellular complexities. For instance, water can move freely across the all‐aqueous interfaces under the change of osmotic pressure in the system, resulting in altered volumetric ratio of the two phases as well as shifting the interfaces dynamically.^79^, ^91^ The osmosis‐induced water exchange through the interfaces can lead to dynamic transformation of single all‐aqueous emulsion into double or triple emulsion with multiple compartments, as demonstrated in **Figure** **3**. Each compartment is enriched with distinct component, similar to the intracellular process of compartmentalization through LLPS.^126^, ^127^

### 2.3 Partitioning and Compartmentalized Reactions

**Figure 4:**
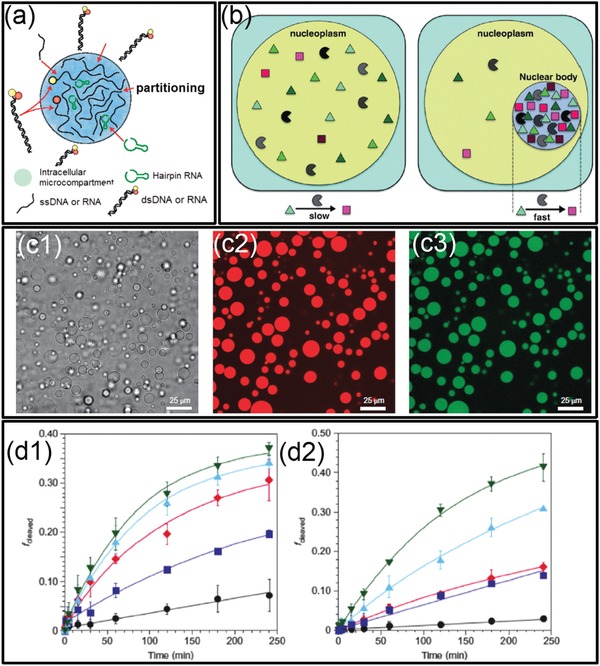
Partitioning and compartmentalized reactions in intracellular LLPS and all‐aqueous emulsions. a) Schematics of the biomolecular partitioning‐assistant intracellular compartmentalized reactions. b) Schematics illustrating the improved rate of the partitioning‐assistant compartmentalized reaction. c) Fluorescent microscope images showing RNA partitions into dextran‐rich phase droplets of ATPS: c1) transmitted light; c2) dextran fluorescence; c3) RNA fluorescence. Scale bars: 25 µm. d) Accelerated RNA cleavage rate, illustrated as the fraction of RNA substrate strands cleaved versus time. a) Reproduced with permission.^133^ Copyright 2016, Springer Nature. b,d) Reproduced with permission.^136^ Copyright 2017, Elsevier. c) Reproduced with permission.^137^ Copyright 2012, Springer Nature.

As facilitated by macromolecular crowding and their interfaces, the interiors of cells can be divided into microcompartments in which specific biological processes are executed and coordinated, including RNA cleavage,^128^ DNA sequestration,^129^ ATP production,^130^ and lipid generation.^131^ To perform these compartmentalized reactions, biomolecules must distribute, react, and redistribute selectively between different microcompartments by equilibrium partitioning.^110^ For instance, in the membraneless organelles with a protein‐rich aqueous compartment surrounded by an aqueous intracellular medium, biomolecules can selectively partition to the protein‐rich phase and exchange with the intracellular cytoplasm, and vice‐versa, facilitating different compartmentalized reactions, such as RNA accumulation within the nucleolus, and preribosomal particles exported from the nucleoplasm to the cytoplasm,^132^ as shown schematically in **Figure 4**a.^133^ Moreover, the partitioning of biomolecules can improve the compartmentalized reaction rates due to colocalization of reactants and the resultant favorable reactive environment (e.g., pH or dielectric constant).^134^, ^135^ It is widely speculated that biological processes involving many sequential reactions performed by different enzymes could be accelerated by partitioning‐assistant compartmentalized reactions, as illustrated schematically in Figure 4b.^134^, ^136^

Recently, plenty of new applications taking advantage of the preferential partitioning of ATPS have emerged and bring the scientific importance of all‐aqueous emulsions to a high level. Specifically, inspired by the anticipated benefits of partitioning‐assistant compartmentalized reactions, all‐aqueous emulsions have been investigated as prime candidates for developing protocells, bioreactors, artificial liquid organelles, and other bioinspired intracellular mimics. For example, single‐stranded RNA and DNA as well as native proteins usually partition to the dextran‐rich phase because of favorable interactions with the dextran‐rich phase which is more hydrophilic, making dextran‐rich phase the suitable location for executing intracellular compartmentalized reactions, such as the RNA cleavage reaction shown in Figure 4c.^137^ Moreover, the reaction rates can also be significantly improved when conducting the reaction in all‐aqueous emulsions compared with in single‐phase aqueous solution. For example, when the RNA cleavage reaction is conducted in ATPS, the reaction rate can be accelerated by 66‐folds due to the partitioning of reactants and catalyst into the droplet phase (Figure 4d),^136^ indicating that LLPS plays a vital role in promoting the origin of early life and protocell formation.

## 3 Cell‐Inspired All‐Aqueous Microfluidics: Discovery of Controllable Artificial LLPS

The merits of all‐aqueous emulsions lie in their capacity to build a compartmentalized aqueous environment resembling membraneless organelles, suggesting the feasibility of their use as templates for developing cell‐inspired biomaterials. Though all‐aqueous emulsions can be generated with traditional emulsifying techniques, such as vortex mixing, homogenization, and dispensing process,^138^, ^139^, ^140^ the size and structure of all‐aqueous emulsions can hardly be controlled, not to mention the mimicry of the highly complex intracellular LLPS and the resultant biomaterials. With recent progress in microfluidic technology,^141^ all‐aqueous emulsions can be precisely manipulated in microchannels, allowing the production of droplets and jets with designable structures and tailored sizes

### 3.1 Cell‐Inspired All‐Aqueous Microfluidic Droplet Generation

Droplets act as the basic function unit for forming intracellular LLPS and constructing compartmentalized environment; therefore, to facilitate the in‐depth mimetic of the intracellular LLPS in vitro, the physicochemical property of all‐aqueous emulsion droplets, such as the size, shape, and composition, should be well controlled. However, controllable formation of all‐aqueous droplets cannot be easily achieved in typical microfluidic channels since their interfacial tensions are ultralow.^122^, ^123^, ^124^ Specifically, in the typical co‐flowing or flow‐focusing microfluidic device, the ultralow interfacial tension limits the nozzle‐confined breakup of water‐in‐water jet,^142^ leading the two aqueous phases to form a liquid jet which is separated by a long and straight interface rather than break up into uniform droplets spontaneously.^77^ Therefore, to achieve the control over the quantitative generation of all‐aqueous droplets in microfluidic channels, methods incorporating a variety of external forces have been developed.

#### 3.1.1 Hydrodynamic Instability

**Figure 5:**
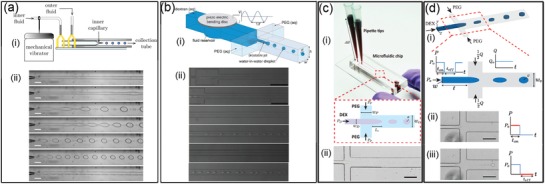
All‐aqueous emulsion droplets generated by utilizing hydrodynamic instability. Schematics of the microfluidic device with perturbing by a) a mechanical vibrator and b) a piezoelectric actuator or by c) hydrostatic pressures and d) pulsating inlet pressures. All the scale bars are 200 µm. a) Reproduced with permission.^79^ Copyright 2012, AIP Publishing. b) Reproduced with permission.^145^ Copyright 2010, Royal Society of Chemistry. c) Reproduced with permission.^143^ Copyright 2016, American Chemical Society. d) Reproduced with permission.^144^ Copyright 2015, Royal Society of Chemistry.

Since breakup of all‐aqueous liquid jet is delayed by the low growth rate of instability driven by the ultralow interfacial tension, external mechanical vibration is introduced into microfluidic channels to generate stronger corrugations on the surface of all‐aqueous liquid jets. By perturbing the pumping pressure applied to the inner dispersed aqueous phase with a mechanical vibrator or a piezoelectric actuator,^91^ monodisperse all‐aqueous droplets can be prepared with tunable sizes and uniform size distribution, as shown schematically in **Figure 5**a,b. The size and generation rate of droplets can be tuned by changing the frequency and amplitude of perturbation quantitatively; for instance, the droplet size can be quantitatively changed, following the relationship *d* = (6*Q*_in_/*f*)^1/3^, where *Q*_in_ is the flow rate and *f* is the frequency of the applied perturbation, as demonstrated by the microscopic images in Figure 5a,b. A technique using hydrostatic and pulsating inlet pressures has also been developed by modifying a polydimethylsiloxane‐based microfluidic device.^143^ The fluid to be injected into microfluidic device is filled into pipette tips as fluid columns at the inlets, which introduces low speed flows to the flow‐focusing junction device, as shown in Figure 5c. The droplet size varies with the interfacial tension and viscosity of the aqueous fluids and the device height at the fluid inlets. To generate all‐aqueous droplets with uniform and tunable size,^144^ the pulsating pressure is controlled by a pneumatic solenoid valve with variable on–off pressure cycles that lets the dextran‐rich phase enters through. The PEG solution enters the flow focusing junction through the cross channels at a fixed flow rate. The on–off cycles of the applied pressure, combined with the fixed flow rate cross flow, contribute together to break the ATPS jet into droplets, as shown in Figure 5d.

#### 3.1.2 Electric Hydrodynamic Instability

**Figure 6:**
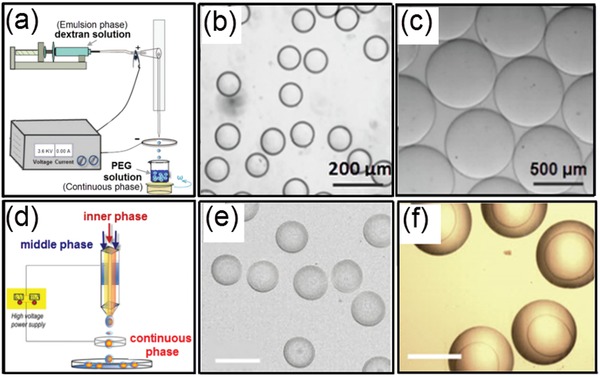
Generation of all‐aqueous emulsion droplets with tunable sizes and strictures via electrospray. a) Schematics of the electrospray setup for generating single emulsion droplets. b,c) Optical microscope images showing the electrosprayed all‐aqueous droplets with tunable sizes. d) Schematics of the formation of core–shell structured all‐aqueous droplets. e,f) Optical microscope images showing core–shell structured all‐aqueous droplets. a–c) Reproduced with permission.^93^ Copyright 2015, American Chemical Society. d–f) Reproduced with permission.^77^ Copyright 2013, AIP Publishing.

Besides taking advantage of hydrodynamic instability, electrohydrodynamic spray is used as another technique for generating monodisperse all‐aqueous emulsion droplets, as illustrated in **Figure 6**a–c.^93^ Specifically, a dextran‐rich liquid jet is charged positively and breaks up into droplets at the end of a capillary nozzle. These charged droplets are forced to go through a negatively charged metallic ring, which is positioned beneath assures that all droplets can pass through the ring before collection in the PEG‐rich continuous phase. The quantitative control over the size and generation rate of droplets can be achieved by adjusting the applied electric field intensity. Apart from single emulsion droplets, core–shell structured droplets can also be generated by the all‐aqueous electrospray technique.^77^ As schematically shown in Figure 6d, a capillary with a tapered nozzle is coaxially inserted into another tapered capillary, forming a co‐flowing geometry. Two immiscible aqueous phases are separately injected into the two capillaries, forming a water‐in‐water liquid jet. The co‐flowing jet is charged from the outer liquid phase, and the composite jet is pumped to go through the counter electrode by electrostatic forces. Upon breakup of the jet, core–shell structured droplets finally fall into another continuous phase, as shown by the microscope images in Figure 6e,f. The relative sizes of the core and shell of the droplets can be tuned by changing the flow rate ratios in the capillaries.

### 3.2 Cell‐Inspired All‐Aqueous Microfluidic Liquid Multiphase Jet Generation

**Figure 7:**
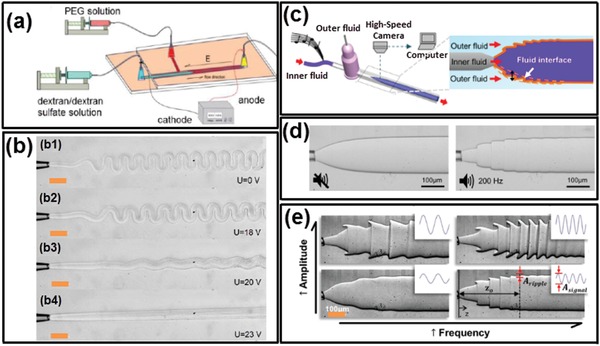
Manipulation of all‐aqueous microfluidic liquid jets. a) Schematics of the microfluidic device for manipulating all‐aqueous jets by electrical charging at applied DC voltages. b) Optical microscope images of all‐aqueous jets under different electrical voltage; scale bars: 200 µm. c) Schematics of hearing by the proposed “interfacial ear” of the all‐aqueous liquid jets. d) Visualization of the deformation of the all‐aqueous interfaces in response to the applied vibration; left: no external vibration is applied; right: a musical note of 200 Hz is applied by a loudspeaker. e) Optical microscope images of the all‐aqueous liquid jets as the frequency and the amplitude of the mechanical wave. a,b) Reproduced with permission.^78^ Copyright 2013, Royal Society of Chemistry. c–e) Reproduced with permission.^151^ Copyright 2014, Springer Nature.

Besides all‐aqueous emulsion droplets, all‐aqueous liquid multiphase jets have been widely discussed and used in printing 1D fiber, 2D membrane, and 3D scaffold, with great potentials to be used in various fields, such as cell culture and patterning, membrane filtration, and tissue engineering.^146^, ^147^, ^148^, ^149^ In core–sheath structured all‐aqueous jets, the slow growth of Rayleigh–Plateau instability driven by the ultralow w/w interfacial tension often results in a long and stable jet. When the viscosity ratio of the outer to inner fluids, η_out_/η_in_ ≪ 1, the inertial force of the inner fluid dominates the deformation of the jet interface. Upon being injected into a dilated channel geometry, a highly viscous inner jet decelerates but can hardly expand in diameter as a nonviscous jet does. Instead, the viscous inner jet forms a periodic folding structure to minimize the dissipation/friction energy.^150^ Changing the flow rate ratio of the inner to outer fluids cannot significantly change the diameter of the folded jet. To manipulate the diameter of the viscous jet, a DC power supply is connected to the inlet of the inner fluid and the outlet of the outer fluid.^78^ At sufficiently high voltages, the folding instability of the viscous jet is suppressed, so the jet morphology and diameter can be easily adjusted by varying the applied voltage, as shown in **Figure 7**a,b. When the viscosity ratio of the outer to inner fluids, η_out_/η_in_ ≫ 1, the inner jet can be easily inflated or deflated by the fluctuation of pumping pressure or mechanical agitation from the surrounding environment.^80^ Taking advantage of this property, variable pumping pressure with programmed perturbating amplitude and frequency have been applied to oscillate the jet phase, as schematically illustrated in Figure 7c. The inner liquid jet inflates or deflates in response to the applied perturbation frequency and amplitude, as shown in Figure 7d,e.^151^ This approach can convert the mechanical wave of sound/music into dynamic fluidic structures, which is useful for detection of the sound signal. In summary, the all‐aqueous jet exhibits usually high sensitivity to the external mechanical or electrical fields that cannot be easily found in transitional water/oil‐based microfluidic systems, attributed from the ultralow interfacial tension of the aqueous two‐phase system.

## 4 All‐Aqueous Microfluidic Droplet Templated Biomaterials: Assembly of Artificial LLPS I

Taking advantages of various cell‐inspired fluidic structures created by all‐aqueous microfluidics and intrinsic characteristics of LLPS, all‐aqueous droplets can be used as templates for shaping and structuring biomaterials. In this section, we show the conversion from droplets into advanced biomaterials using different approaches.

### 4.1 Chemical Polymerization

#### 4.1.1 Chemical Reaction‐Induced Polymerization

**Figure 8:**
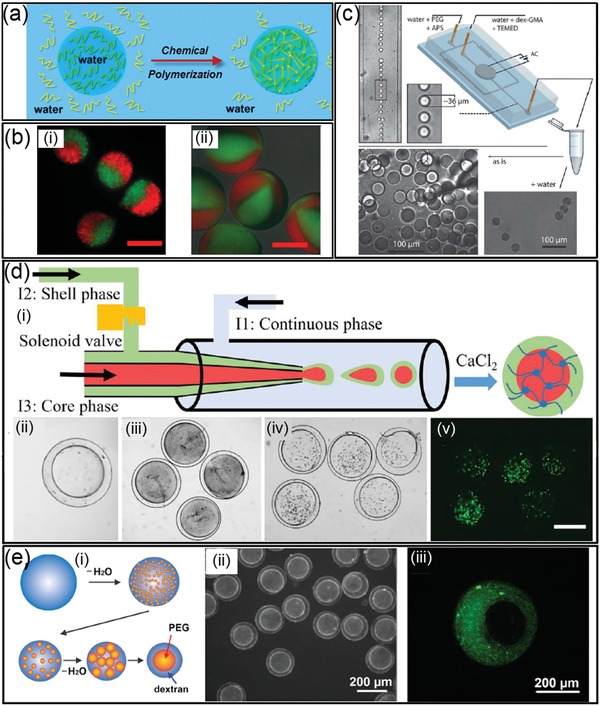
Chemical polymerization to convert all‐aqueous droplets into composite architectures. a) Schematics of the polymerization of all‐aqueous droplets into hydrogel particles. b) Fluorescence microscope images of generated multicompartment Ca‐alginate particles; scale bars: 200 µm. c) Dex‐GMA hydrogel microsphere formation from aqueous two‐phase droplets. d) Schematics of the all‐aqueous phase microfluidic system for the fabrication of calcium alginate microcapsules: ii,iii) Optical microscope images of morphology of the capsules fabricated at different core phase flow rates; iv,v) fluorescent microscope images of cell‐laden calcium alginate microcapsules; scale bars: 200 µm. e) Schematics of the transition of emulsion structures from w/w single emulsion to w/w/w double emulsion: ii,iii) Collagen selectively partitions into the dextran‐rich shell phase (pH 6), as confirmed by the green fluorescence of FITC‐collagen emitting from the fabricated collagen microcapsules. b) Reproduced with permission.^153^ Copyright 2013, AIP Publishing. c) Reproduced with permission.^145^ Copyright 2010, Royal Society of Chemistry. d) Reproduced with permission.^92^ Copyright 2019, American Chemical Society. e) Reproduced with permission.^93^ Copyright 2015, American Chemical Society.

Various chemical reactions have been utilized to convert all‐aqueous droplets into hydrogel microparticles. For instance, monomers and crosslinking agents are added separately from the dispersed and continuous phase of all‐aqueous emulsions,^81^ as illustrated schematically in **Figure 8**a. Examples include calcium alginate hydrogel microparticles fabricated by loading sodium alginate in the droplets and calcium chloride in the continuous phase. As small molecules of calcium chloride can easily diffuse across the aqueous interface, the crosslinking reaction is initiated at the droplet interface and propagates throughout the whole droplet phase. Clotting of microfluidic channels can be prevented by optimizing the channel geometry, localizing the release of crosslinkers in the droplet phase, or separating the droplet and continuous phases through electrospray.^152^ For instance, methacrylate derivatized dextran (dex‐GMA) is designed as photocurable monomers which strongly partitions in the droplet phase. As a result, the polymerization is confined within the droplet phase once the radical initiator ammonium peroxydisulfate (APS) in the PEG‐rich phase diffuses into the droplet phase, as shown in Figure 8c.^145^ Using multichannel microfluidic devices, hydrogel particles can be prepared with multicompartments, and each compartment is loaded with identical or different components (Figure 8b).^153^ When different types of cells or growth factors are encapsulated in different compartments, the resultant multicompartment particles can be used for studies of noncontact cell–cell interactions.^153^

Moreover, the crosslinking reaction between sodium alginate with CaCl_2_ can be adopted to precipitate the middle phase of the water‐in‐water‐in‐water (w/w/w) emulsion and generate microcapsules with core–shell structures. As schematically illustrated in Figure 8d, w/w/w emulsion droplets are created in a microfluidic channel with sodium alginate dissolved in the middle aqueous phase. The generated all‐aqueous droplets are collected in a CaCl_2_ solution, where the middle aqueous phase is crosslinked into hollow shells of microcapsules, as shown in Figure 8d. Furthermore, the fabricated microcapsules can be used as promising vehicles for cell encapsulating and culturing, as demonstrated by the fluorescent images in Figure 8d.^92^ Moreover, gelation of phase‐separating droplets formed at the different time points of LLPS enables engineering the internal structures of the resultant capsules, as well as regulating the kinetics of the biochemical reactions in the droplet‐based bioreactors.^154^, ^155^ As illustrated schematically in Figure 8e, collagen is first loaded to the droplets dissolved with dextran and PEG before the start of phase separation. Upon triggering the phase separation of the droplet phase by an osmotic dehydration, the collagen precursor spontaneously partitions to the dextran‐rich shell phase. By adding dextran sulfate as crosslinker of collagen, the shell phase can be quickly immobilized. At the early stage of phase separation, fast gelation of the shell leads to the formation of capsules encapsulating multiple subcompartments of PEG‐rich droplets with polydisperse size distribution, as shown in Figure 8e.^93^ At a later stage of phase separation, the gelation of the shell results in monodisperse capsules with single cores inside.

#### 4.1.2 Photoinitiated Polymerization

**Figure 9:**
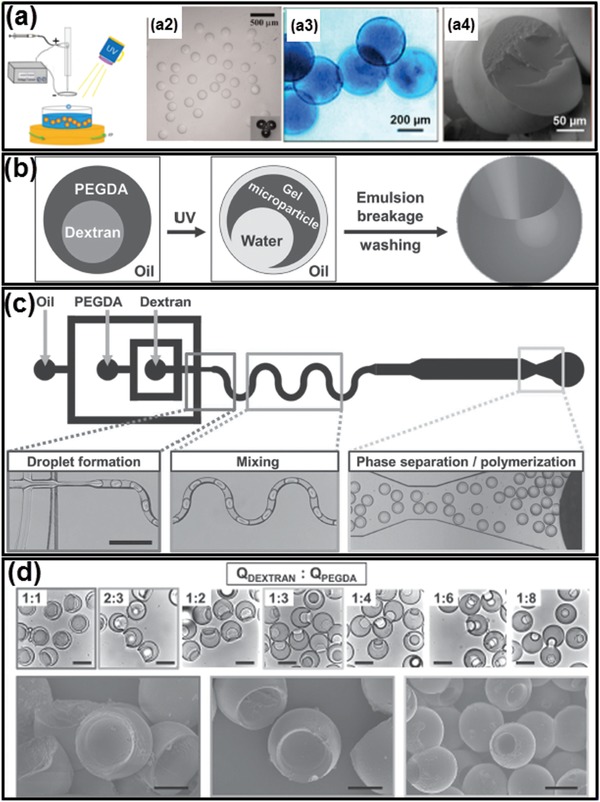
Photoinitiated polymerization converts all‐aqueous droplets to complex biomaterials. a) Schematics and microscope images of photo‐crosslinked PEGDA particles generated from ATPS droplets. b) Schematics of UV‐induced droplet polymerization accompanied by shrinkage of the PEGDA shell. c) Schematics of a microfluidic device generating dextran‐in‐PEGDA emulsion droplets; scale bars: 200 µm. d) Optical microscope images of microparticles generated with a socket shape. The size of open sockets can be tuned by the changing flow rate ratios of dextran and PEGDA (upper row); scale bars: 80 µm. Scanning electron microscope (SEM) images of selected samples (lower row); scale bars: 30 µm. a) Reproduced with permission.^93^ Copyright 2015, American Chemical Society. b–d) Reproduced with permission.^158^ Copyright 2012, Wiley‐VCH.

In applications that require fast and massive production of hydrogel with predesigned shape, photopolymerization enables light‐induced gelation of all‐aqueous droplets within seconds. Photocurable monomers, such as poly (ethylene glycol) diacrylate (PEGDA), are used as the dispersed phase, and the continuous phase could be either dissolved with concentrated dextran or K_3_PO_4_. When irradiated by ultraviolet (UV) light illumination, the PEGDA droplets are crosslinked into hydrogel particles via free radical polymerization, as shown in **Figure 9**a. If the UV light is partially blocked by a photomask with engraved pattern, the resultant particles can be made into different shape or labeled with barcodes. More complexed architecture can also be designed by changing the liquid structure of all‐aqueous emulsions. For example, socket‐like hydrogel particles are obtained when using w/w/o double emulsion templates consisting of dextran‐rich core and a photopolymerizable PEGDA shell, as illustrated in Figure 9b,c. The socket‐like shape of the emulsion templates is driven by the tendency to minimize the interfacial energy of the complexed emulsion system.^156^, ^157^ The interfacial tension between PEGDA and the oil, γ_PEGDA‐oil_, is smaller than that between dextran‐rich phase and oil, γ_dextran‐oil_, so the dextran‐in‐PEGDA‐in oil emulsion droplets are stable. However, upon UV exposure, the all‐aqueous droplets undergo shape‐transformation, yielding PEGDA microgel particles containing an open socket. The transformation is induced by contraction of the PEGDA shell during polymerization, which squeezes out dextran (and FITC–dextran) molecules from the droplet core into the PEGDA shell, as shown by Figure 9d. After PEGDA is fully polymerized, the dextran‐rich phase is only partially trapped inside the PEGDA polymer network, yielding dextran/PEGDA hydrogel microparticles with an open socket. Moreover, the degree of the open‐socket can be tuned by changing the flow rate ratios between the PEGDA and dextran phase in the microfluidic channels, as shown in the images in the upper row in Figure 9d.^158^

### 4.2 Physical Conversion

#### 4.2.1 Thermal‐Induced Gelation

**Figure 10:**
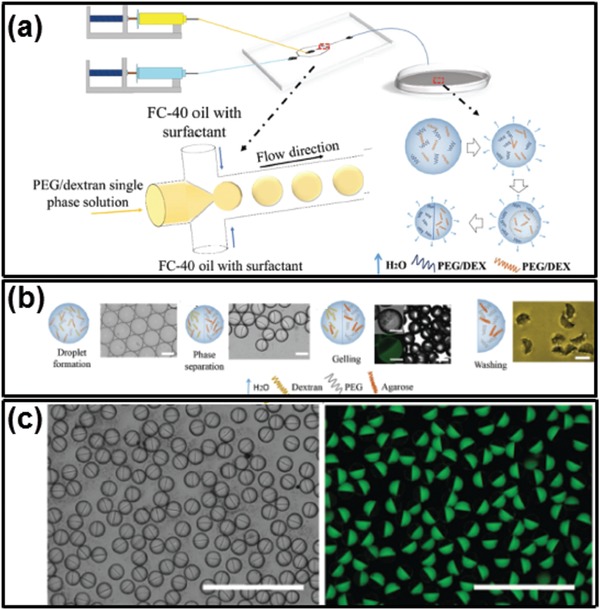
Thermal‐induced gelation of all‐aqueous droplets into Janus particles. a) Schematics of the fabrication process. b) Schematics and optical microscope images illustrating the fabrication of sickle‐shaped particles templated from Janus droplets; scale bars: 50 µm. c) Optical and fluorescence microscope images of the Janus droplets and particles; FITC–dextran is used to label dextran‐rich phase. Scale bars: 100 µm. Reproduced with permission.^162^ Copyright 2017, Wiley‐VCH.

Many biomolecules and synthetic polymers undergo liquid–solid phase transition in response to temperature changes. Examples include collagen, matrigel, agarose, and agar.^159^, ^160^, ^161^ Loading these biomolecules into all‐aqueous emulsions provides a simple approach to prepare hydrogel particles via temperature control.^162^ As an example, when agarose is loaded into Janus droplets of dextran/PEG‐in‐oil emulsion, the agarose partitions into the PEG‐rich continuous phase. Once the temperature drops down below 17 °C, the PEG‐rich phase containing liquid agarose will be solidified.^162^ After washing with water, the dextran‐rich compartment will be dissolved, resulting in sickle‐shaped particles, as shown in **Figure** **10**. The size of the particles can be adjusted by tuning the flow‐rate ratios between the outer and inner phases.

**Figure 11:**
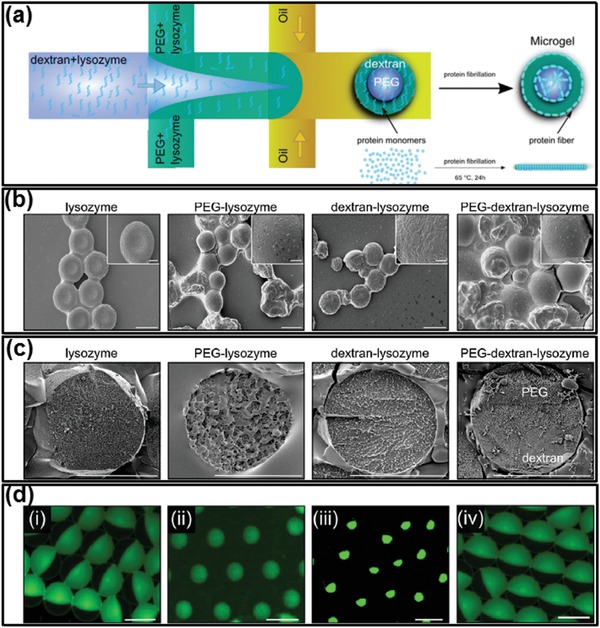
Thermal‐induced fibrillization of protein monomers and gelation of amyloid networks. a) Schematics showing the fibrillation of lysozyme monomers and the gelation of ATPS droplets when increasing the temperature. b) SEM and c) cryo‐SEM images of lysozyme microgel particles forming using dextran–PEG ATPS droplets. Scale bars: 20 µm. d) Fluorescent microscope images of dextran‐core with PEG‐shell lysozyme microgels with different core and shell sizes; scale bars: 20 µm. Reproduced with permission.^163^ Copyright 2014, Wiley‐VCH.

Temperature variations can also initiate polymerization of biomolecules such as proteins in all‐aqueous emulsions.^163^ Monomeric proteins, such as lysozyme, are first dissolved in the dextran and PEG solution separately. The dextran/lysozyme and PEG/lysozyme mixture are then separately injected into co‐flowing microfluidic channel as the innermost and middle phase, and the outer phase is fluorinert oil. After formation of dextran‐in‐PEG‐in‐oil double emulsion droplets, the lysozyme monomers are mainly distributed in the dextran‐rich phase. As the temperature increases to 60 °C, the protein monomers are unfolded and start to polymerize into amyloid nanofibrils with β‐sheet structure. Further incubation of the amyloid fibrils enhances the intermolecular forces and yields amyloid gel, as shown in **Figure 11**b,c. The size and geometry of the amyloid microgels can be tuned by regulating the relative flow rates, as shown in Figure 11d. Using droplet templates also allows “time‐stretched” visualization of the kinetics in phase transition of proteins and nuclear acids, which is an important tool for understanding the assembly and dynamic behaviors of biomacromolecules.

**Figure 12:**
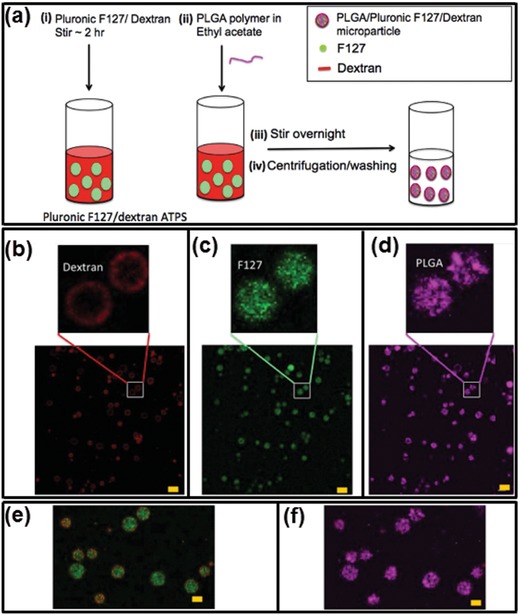
PLGA‐based microparticles fabricated in a temperature‐dependent self‐assembly Pluronic F127/dextran ATPS. a) Schematics of the fabrication process. b–f) Three‐color fluorescent microscope images of prepared microparticles (TRITC‐dextran, red; FITC‐Pluronic, green; Cy5‐PLGA, cyan). The dextran polymers form an outer shell whereas the Pluronic and PLGA polymers are enriched in the core; scale bars: 10 µm. Reproduced with permission.^169^ Copyright 2016, Springer Nature.

In addition, functional copolymers with pH/temperature responsiveness have also been introduced to form LLPS and to design smart particles/capsules.^164^, ^165^, ^166^ An simple example of this copolymer is Pluronic F127, which is a commercialized PEG–PPG–PEG copolymers with a LCST around 37 °C.^167^, ^168^ Pluronic F127/dextran aqueous two‐phase system has been adopted as templates to fabricate poly(lactic‐co‐glycolic acid) (PLGA) microparticles. PLGA is first dissolved in ethyl acetate before getting emulsified in ATPS mixture of dextran/Pluronic F127 solution through stirring at 37 °C overnight. After the temperature is dropped to 4 °C, the Pluronic F127/dextran ATPS is oversaturated; as a result, PLGA is precipitated out of the aqueous mixture and self‐assembles to microparticles, as illustrated schematically in **Figure 12**a and the fluorescence microscope images in Figure 12b,d.^169^ The incorporation of both hydrophobic and hydrophilic components into the multiphase particles allows programmed design of temperature‐sensitive properties for controlled drug release.

#### 4.2.2 Osmosis‐Induced Solidification

**Figure 13:**
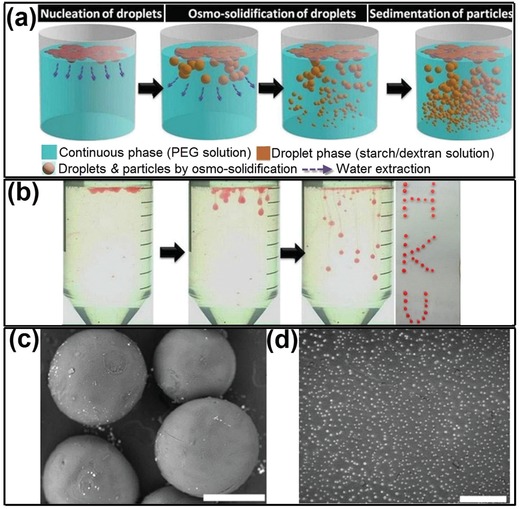
“Osmo‐solidification” converts all‐aqueous droplets to solidified particles. a) Schematics of the osmo‐solidification process. b) Digital photos of the osmo‐solidification process and the final particles dyed by Nile red. The SEM image of c) osmo‐solidified starch particles; scale bar: 400 µm, and d) dextran nanoparticles; scale bar: 4 µm. Reproduced with permission.^76^ Copyright 2016, Royal Society of Chemistry.

Similar to intracellular LLPS, the dynamic interface of all‐aqueous emulsions allows transfer of water between the emulsion and continuous phases.^170^, ^171^ Gelation of all‐aqueous droplets can be conveniently induced by condensing the droplet phase preloaded with gel‐forming precursors. The condensation can be simply realized by using the osmolarity gradient across the droplet and continuous phases. Specifically, if the solute concentrations in the ATPS are not in phase‐equilibrium, an osmotic pressure gradient temporarily exists across w/w interface, and the chemical potential gradient drives the transfer of water from one aqueous phase to the other. Particularly, when the two aqueous phases are far away from phase‐equilibrium state, the hypertonic continuous phase (e.g., 30 wt% PEG solution, Mw = 4000) can continuously suck water out of the hypotonic emulsion phase (e.g., 8 wt% dextran, Mw = 500 000) until the droplet phase is condensed into gel particles. This “osmo‐solidification” approach can be used for encapsulation of enzymes and preservation of their catalytic activity, as schematically illustrated and experimentally demonstrated in **Figure 13**a–d. This technique offers a mild environment for encapsulation and drying of active proteins without imposing evaporation‐induced stress at water–air interface. Therefore, the stability of the encapsulated proteins can be better preserved compared to evaporation in air and lyophilization.^76^

## 5 All‐Aqueous Interface Templated Biomaterials: Assembly of Artificial LLPS II

Inspired from the lipid bilayer of cell membrane, interfaces of all‐aqueous emulsions have been employed as 2D templates for fabrication of vesicles composed of a few macromolecular layers, including liposomes,^94^, ^172^ polymersomes,^173^ and colloidosomes.^174^, ^175^ Compared to the water–oil interface, the dynamic aqueous–aqueous interface has an ultralow interfacial tension, which gives rise to challenges and opportunities in macromolecular assembly at liquid–liquid interface.^95^ In recent years, the fundamental understanding on the macromolecular assembly at aqueous–aqueous interfaces has been propelled, and new surfactant systems have been experimentally developed to stabilize all‐aqueous emulsions and to fabricate vesicles in near‐physiological environments.^131^, ^176^, ^177^ It is worth stating that the distribution of complexes at the liquid–liquid interfaces is heterogeneous. Particularly, when the adsorption energy is relatively high, large nanoparticles and nanofibrils can be irreversibly trapped at the aqueous–aqueous interface with certain contact angles, leading to the heterogeneous distribution of solid complexes across the liquid–liquid interfaces. On the contrary, when the adsorption energy is relatively low, small particles and molecules cannot be trapped at the aqueous–aqueous interface. Reagents formulating the complexes can diffuse across the liquid–liquid interfaces and react with compounds in the other aqueous phase. The resulting complexes at the liquid–liquid interface are also heterogeneously distributed, depending on the partitioning coefficient of the complex in the ATPS.

### 5.1 Stabilization of Aqueous–Aqueous Interface

**Figure 14:**
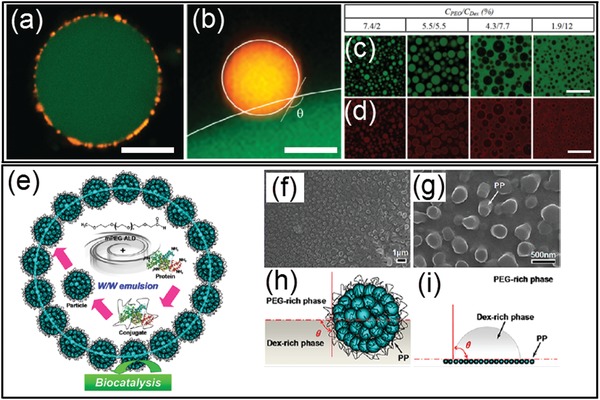
a–d) Physical adsorption of small particles at the all‐aqueous interfaces: a) A CLSM image of a dextran‐rich droplet (green) coated with latex particles (yellow) in the PEO phase; scale bar: 20 µm. b) A CLSM image showing a latex particle (yellow) trapped at the interface between the dextran‐rich (green) and PEG‐rich phases. The surface of the particle and the dextran/PEO interface are indicated by white lines and the contact angle is shown; scale bar: 0.5 µm. c,d) CLSM images showing protein particles (red) stabilized PEO/dextran(green) mixtures at different weight ratios. Scale bars: 80 µm. e–i) Anchor of the polymer–protein conjugate particles at the all‐aqueous interfaces: e) Design of the polymer–protein conjugate particles with biocatalytic activity for the stabilization of all‐aqueous interfaces. f,g) SEM images showing morphology and wettability of mPEG–BSA conjugate particles at the all‐aqueous interfaces. h) Schemes of a conjugated particle at the PEG/dextran interfaces. i) Measurement on the surface wettability of the conjugated particle. a,b) Reproduced with permission.^183^ Copyright 2012, American Chemical Society. c,d) Reproduced with permission.^184^ Copyright 2013, American Chemical Society. e–i) Reproduced with permission.^185^ Copyright 2017, American Chemical Society.

Unlike water–oil interfaces, interfaces of the all‐aqueous emulsions show poor discontinuity in polarity, leading to the reduction of the driving force for the interfacial adsorption of amphiphilic surfactants.^178^ On one hand, the thermal energy to trap a surfactant molecule/particle at the aqueous–aqueous interface is directly proportional to the interfacial tension, so the low interfacial tension of ATPS leads to a low driving force for attachment of surfactant molecules/particles.^179^ On other hand, small particles dynamically enter and detach from the aqueous–aqueous interface, which is driven by Brownian motion, resulting in reversible and unstable interfacial adsorption. Particularly, when the kinetic energy of Brownian motion is relative higher than the thermal energy to trap the surfactant at the aqueous–aqueous interface, surfactants cannot be irreversibly trapped at the interface, and the neighboring droplets will eventually coalesce.^180^, ^181^, ^182^ For instance, latex particles with a radius no less than 0.1 µm can be irreversibly trapped at the interfaces of the polyethylene oxide (PEO)/dextran ATPS with interfacial tensions down to 10^−6^ N m^−1^, as shown by the confocal laser scanning microscope (CLSM) images in **Figure 14**a,b.^183^ On the contrary, nanoparticles of polystyrene with an average radius of few tens of nanometers cannot be trapped at the aqueous–aqueous interface. This phenomenon implies that the absorption energy of the small particles has to be larger than the kinetic energy imposed by thermal activation. Besides, heat‐denatured protein particles have been shown to stabilize the aqueous–aqueous interfaces. Contrary to small native proteins, aggregated protein particles induced by heat‐denature process can form a monolayer at the all‐aqueous interfaces and inhibit fusion of neighbored emulsion droplets. With a sufficiently large concentration of protein particles and a relatively high aqueous–aqueous interfacial tension, protein particles can successfully stabilize the PEG/dextran interfaces for a few weeks, as demonstrated in Figure 14c,d.^184^ These studies indicate that the presence of intracolloidal forces, such as hydrophobic interaction between heat‐denatured protein particles facilitates the stabilization of all‐aqueous emulsions. Moreover, the surface wettability of surfactant is another important factor that contributes to the stabilization of all‐aqueous emulsion. For instance, while protein particle alone may not sufficient to stabilize all‐aqueous emulsion, the conjugation of PEG to protein particles may improve their interfacial adsorption due to the change in wetting angle, as shown in Figure 14e. The use of protein catalyst enables formation of functional capsules with catalytic activity.^185^

### 5.2 Assembly of Vesicles at All‐Aqueous Interface

#### 5.2.1. Polymersomes

**Figure 15:**
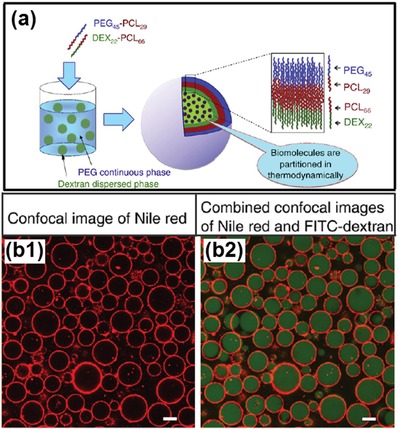
a) Schematics of the preparation procedure and structure of asymmetrical polymersomes. b) The polymersome is composed of asymmetric bilayer membrane conjugated from PEG–PCL and dextran–PCL copolymers; scale bars: 10 µm. a,b) Reproduced with permission.^173^ Copyright 2013, Elsevier.

Adsorption of amphiphilic copolymers at the all‐aqueous interfaces offers a promising approach to interfacial assembly. For instance, two copolymers of the PEG–poly caprolactone (PCL) and the dextran–PCL are separately added into the PEG‐rich and dextran‐rich phases. Upon the mixing of the two phases, the two copolymers spontaneously aggregate at the all‐aqueous interfaces, forming “polymersomes” as shown in **Figure 15**a,b.^173^ Formation of the compact polymersomes at the aqueous–aqueous interface relies on the PCL moieties, which enhances the hydrophobic interactions between PEG–PCL and dextran–PCL copolymers. The results demonstrate that the assembly of asymmetric giant vesicles is strongly affected by their interactions with the dissolved additives constituting the aqueous phases.

#### 5.2.2. Liposomes

**Figure 16:**
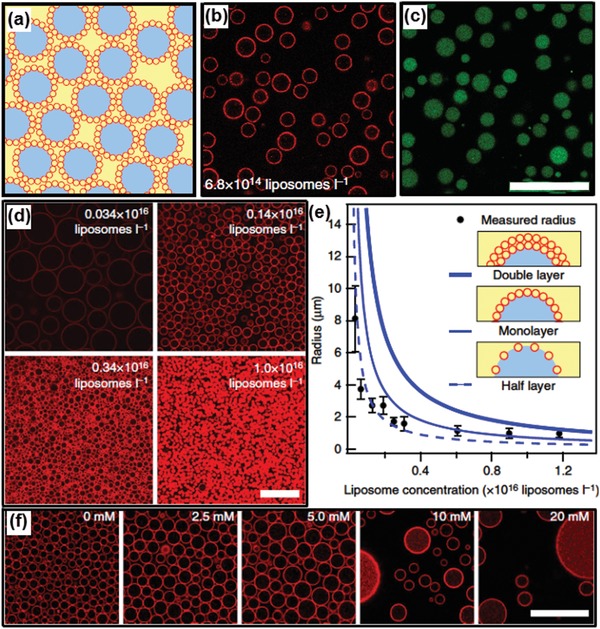
Liposome‐stabilized all‐aqueous interfaces. a–c) Dextran droplets dispersed in PEG continuous phase. a) Schematic illustration (red, liposomes; blue, dextran phase; yellow, PEG phase). b) Fluorescence image showing location of DOPE‐Rhodamine‐labeled liposomes at the aqueous/aqueous interface. c) Fluorescence microscope image showing location of dextran phase labeled with FITC–dextran. d) Fluorescence microscope images show that the size of droplets is inversely correlated with the concentration of liposomes. e) Dependence of stabilized droplet sizes with liposome concentrations. f) Screening of charge leads to emulsion destabilization. At low ionic strength, a reduction in Debye length leads to tighter liposome packing at the interface, increasing the observed droplet size. As ionic strength increases, the liposomes can no longer prevent droplet coalescence. All scale bars are 25 µm. Reproduced with permission.^172^ Copyright 2014, Springer Nature.

The adsorption of typical lipids at all‐aqueous interface is unable to stabilize all‐aqueous emulsions due to the limitation of lipid size and the resultant thermoadsorption energy. Nanosized liposomes, when chemically modified with PEG moiety, can be adsorbed at the all‐aqueous interface with a high affinity. Upon their diameter reaches about 130 nm, the PEGylated liposomes^154^, ^186^ consists primarily of egg phosphatidyl glycerol (PG) and PEG moiety can prevent the coalescence of all‐aqueous emulsion droplets, as demonstrated by the CLSM images in **Figure 16**b,c.^172^ Given that all liposomes are coated on the droplet surface, the number of liposome layers at the droplet interface can be estimated from the concentration of liposomes and total surface area of the droplet stabilized. The average droplet size, which is proportional to the square root of stabilized surface area, is measured as a function of different liposomes concentration.^187^ A higher liposome concentration leads to a decrease in droplet size, as demonstrated in Figure 16d. Based on the observed droplet size and the added concentration of liposomes, liposome coverage at the interface can be estimated. Coating a monolayer of liposomes at the emulsion interface is sufficient to stabilize water/water emulsion, as shown in Figure 16e. The presence of negative surface charge and a low ionic strength facilitates the formation of monolayer, as demonstrated in Figure 16f.

##### 5.2.3. Colloidosomes

**Figure 17:**
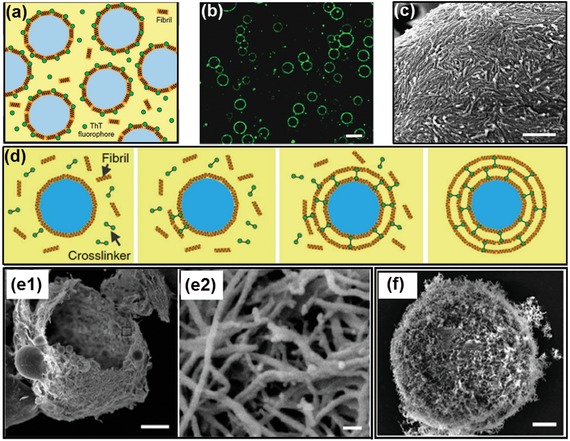
Colloidosomes fabricated from the all‐aqueous interfaces. a) Scheme and b) fluorescence microscope image of ThT‐dyed lysozyme fibrils accumulated at the all‐aqueous interfaces; scale bar: 20 µm. c) An SEM image confirms that lysozyme fibrils deposit as a monolayer at the emulsion interfaces; scale bar: 500 nm. d) Schematics of the formation of protein fibrillosomes by crosslinking fibril‐coated all‐aqueous interfaces. e) SEM images of lysozyme capsules composed of multilayers of fibrils. All‐aqueous interfaces coated with fibril monolayers are used as the seeding templates, and the formation of multilayer fibrils is induced by the fibrillization of lysozyme monomers. Scale bars: e1) 2 µm; e2)50 nm. f) SEM images of fibrillosomes with their shells consisting of amyloid fibrils; scale bars: 2 µm. Reproduced with permission.^174^ Copyright 2016, Springer Nature.

The adsorption energy of different colloids at the aqueous–aqueous interface is influenced not only by their wetting angle but also the geometry of colloidal particles. Spherical colloidal particles are frequently observed to stabilize emulsion when their wetting angle is close to 90°. Other shapes of colloids, such as nanoplates and nanofibrils, may stabilize water/water emulsions at a broader range of wetting angles. To form robust colloidosomes, a microfluidic approach has recently been developed to generate colloidosomes from multilayered protein fibrils, which is termed as “fibrillosomes.”^174^, ^175^ Protein nanofibrils are prepared by incubation and spinning of lysozyme solution at 60 °C, which triggers the amyloidosis of lysozyme monomers.^188^ When suspended in the dextran/PEG ATPS, lysozyme nanofibrils can be efficiently trapped at the w/w interface, while the lysozyme monomers are enriched in the dextran‐rich phase, as demonstrated by the fluorescent images in **Figure 17**b. Due to the high affinity to the fibrils to PEG‐rich phase, the lysozyme fibrils can stabilize dextran‐in‐PEG emulsion but not PEG‐in‐dextran emulsions. To endow the fibril‐stabilized emulsion with exceptional robustness, the existing fibrils trapped at aqueous/aqueous interface is used as protein seeds to cultivate the fibrillization from surrounding solution, until multilayered fibrils are formed at the aqueous/aqueous interface. The multilayered lysozyme fibrils are covalently crosslinked with glutaraldehyde, resulting in 2D fibril networks, as shown by the SEM images in Figure 17e. Compared with noncovalently crosslinked capsules, the covalently crosslinked fibrillosomes are mechanically robust and remain stable after the PEG‐rich continuous phase is replaced with the same solution inside the droplet, as shown schematically in Figure 17d and cryo‐SEM images in Figure 17f. The robustness of this approach enables the collection of monodisperse fibrillosomes templated from water/water droplets through microfluidic devices. Moreover, similar to the dynamic reversibility of the complexation in LLPS, the adsorption of protein fibrils at the w/w interfaces can also be reversibly detached by changing the interfacial tensions of ATPS through diluting, and results in the breaking up of the fibrillosomes.

### 5.3 Chemical Complexation

**Figure 18:**
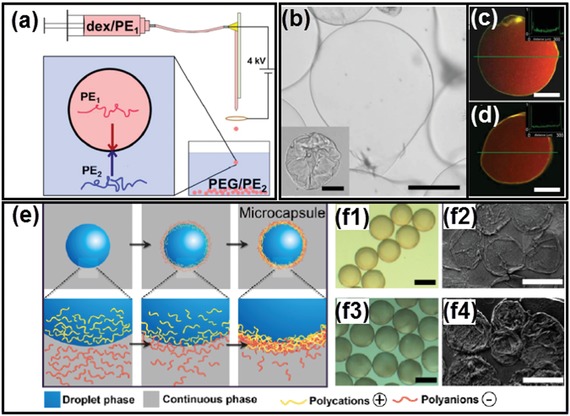
All‐aqueous interfacial complexation via electrostatic interactions. a) Schematics of the electrospray setup. b) A typical capsule made by electrospray with PDADMAC and PSS and dialyzed against water to fully inflate; inset is the capsule as made before dialysis; scale bars: 100 µm. c,d) Formed PDADMAC/PSS microcapsules and encapsulated RD70 dye (red signal) imaged with CLSM; scale bars: 100 µm. e) Schematics of the fabrication process of the assembled PE microcapsules via affinity partitioning. f) Optical microscope and SEM images of the fabricated microcapsules with different PE combinations; scale bars: 300 µm. a–d) Reproduced with permission.^189^ Copyright 2016, American Chemical Society. e,f) Reproduced with permission.^195^ Copyright 2016, American Chemical Society.

**Figure 19:**
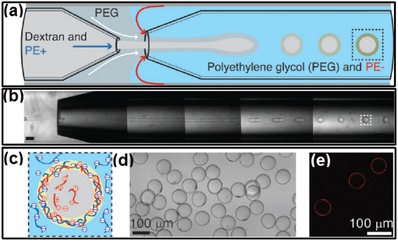
PE complexation in the middle aqueous phase of w/w/w emulsions. a,b) Schematic illustration and optical microscope images of the fabrication process of PE microcapsules. c) Illustration of complexation of two oppositely charged PEs occurring in the middle aqueous phase. d) Optical micrograph of generated microcapsules. e) Fluorescent image of capsule shells labeled with ethidium homodimer. Scale bars: 300 µm. Reproduced with permission.^196^ Copyright 2016, Wiley‐VCH.

Multilayered vesicles can also be fabricated by executing chemical reactions at all‐aqueous interface. A typical approach is to introduce complexation of oppositely charged polyelectrolytes (PEs) or nanoparticles at all‐aqueous interface (**Figure 18**a–d), analogous to the nucleation of membraneless organelles in biological systems.^189^, ^190^, ^191^, ^192^ Similar to the enrichment of RNA in the membraneless organelles, the preferential partitioning of PEs in the ATPS frequently determine the localization of gelation.^193^, ^194^ For instance, if two oppositely charged PEs are added separately from their low‐affinity aqueous phases, the interfacial complexation often results in the formation of microcapsules, as shown in Figure 18e,f.^195^ In contrast, if the same pair of PEs are added from their high‐affinity aqueous phases, the interfacial complexation often leads to the formation of droplet‐templated particles. Besides, by changing the pH values of ATPS, the degree of preferential partitioning of PEs can also be changed, leading to the dynamic control over the formation speed and shell thickness of the resultant PE microcapsules. Moreover, the complexation of two oppositely charged PEs can also induce the solidification of the middle phase of water/water/water emulsion, yielding microcapsules. Specifically, water/water/water emulsions are generated through a co‐flowing microfluidic device. Two PEs are separately dissolved in the outer and innermost aqueous phases, while the middle aqueous phase provides the location for two PEs to react, as shown in **Figure** **19**. Due to the partitioning of PE complexes in the middle phase, gelation of the shell can be achieved. This microfluidic method afford a precise control over the complexation of the PEs,^196^ and the resultant PE microcapsules show sustained release of encapsulated biomolecules. The concept of using w/w/w emulsion as templates is not restricted to the formation of PE capsules, but also applicable to a broad range of engineered polymers with predesigned surface charges and functionalities.^197^

## 6 All‐Aqueous Multiphase Jet Templated Biomaterials: Assembly of Artificial LLPS III

All‐aqueous multiphase jets have been applied as important templates in fabrication of 1D biomaterials, such as hydrogel fibers and woven fabrics, which have great potentials in fields of tissue engineering scaffolds, implantable hydrogel device and biosensors, as well as wearable electronic devices.^198^, ^199^, ^200^, ^201^, ^202^, ^203^

**Figure 20:**
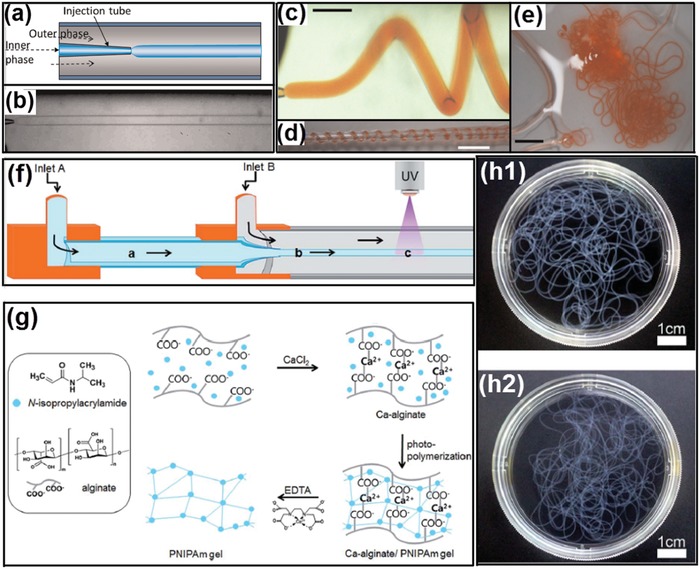
Microfibers fabricated from all‐aqueous liquid jets. a) Schematics of a capillary microfluidic device used in this study. b) Optical microscope image showing a jet of PEG‐in‐dextran in the microfluidic device; scale bar: 200 µm. c) Optical microscope image of a calcium alginate jet; scale bar: 800 µm. d,e) Digital photos of calcium alginate gel fibers encapsulating allurared prepared. Scale bars are 5 and 15 mm in (d) and (e), respectively. f) Schematic diagram of fabrication of temperature‐responsive PNIPAm hydrogel microfibers. g) Procedure for fabricating hydrogel microfibers by the alginate templating method, with EDTA used to dissolve and remove calcium alginate to obtain pure PNIPAm microfibers. h) Digital photos of prepared PNIPAm hydrogel microfibers h1) before and h2) after EDTA treatment. a–e) Reproduced with permission.^81^ Copyright 2012, AIP Publishing. f–h) Reproduced with permission.^204^ Copyright 2015, Royal Society of Chemistry.

Due to the slow growth of Rayleigh–Plateau instability, water‐in‐water jets in co‐flowing microfluidic devices are often maintained continuously without breakup. By separately adding calcium chloride and alginic acid in the dispersed phase of PEG solution and the continuous phase of dextran solution, respectively, the all‐aqueous jets are solidified into microfibers after crosslinking of Ca‐alginate, as shown in **Figure 20**a,b.^81^ The fast gelation of alginate enables encapsulation of small molecules, as demonstrated by the digital images in Figure 20c–e. Moreover, if *N*‐isopropylacrylamide (NIPAm) is incorporated into the jet together with alginate, the subsequent photopolymerization of NIPAm results in the formation of PNIPAm hydrogel, which endows the microfibers with the temperature responsiveness, as shown by the schematics in Figure 20f,g and by the digital photos in Figure 20h.^204^

**Figure 21:**
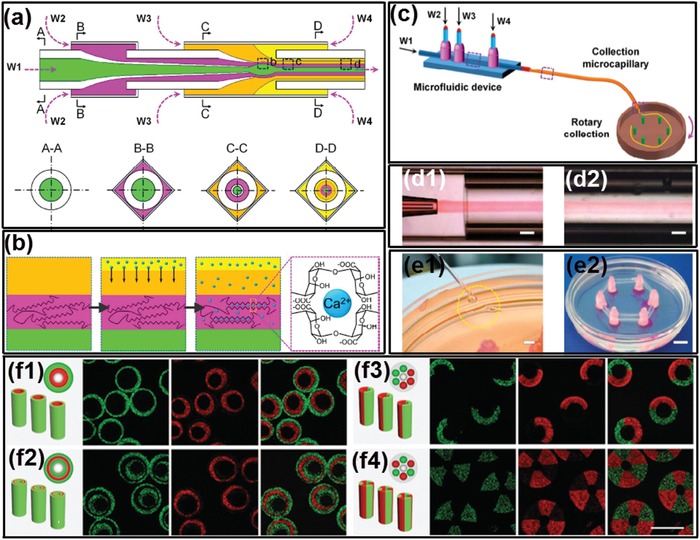
Continuous microfluidic generation of all‐aqueous microfibers with complex structures. a) Schematics of the coaxial microfluidic device for hollow microfiber fabrication. b) Schematics of the fabrication procedure via crosslinking in the sheath flow. c) Illustration showing the continuous microfiber generation and collection. d) High‐speed snapshots showing the generation of d1) cylindrical jet and d2) the resultant hollow microfibers; scale bars: 20 µm. e) Digital photos showing the rotating collection of hollow microfibers. Scale bars: e1) 2 mm; e2) 1 cm. f) Schematic illustrations of the microfibers with multicompartment and their corresponding microfluidic injection channels, and the cross‐sectional CLSM images of the microfibers; scale bars: 200 µm. a–e) Reproduced with permission.^205^ Copyright 2016, Royal Society of Chemistry. f) Reproduced with permission.^206^ Copyright 2014, Wiley‐VCH.

The generation of all‐aqueous multiphase jets in complexed microfluidic techniques provides liquid templates for preparation of fibers with high structural complexity. For example, hollow Ca‐alginate microfibers can be generated by using four aqueous solutions as the aqueous phases as illustrated in **Figure 21**a,b.^205^ The microfibers are continuously synthesized and then collected in a Petri dish by using a roller. The whole process is illustrated in Figure 21c. After washing with deionized water, hollow Ca‐alginate microfibers can be successfully obtained, as shown in Figure 21d,e. Moreover, microfibers containing multicompartments are generated by novel capillary microfluidics with tailored capillary tips and microchannel design in the microfluidic setup. The resultant microfibers possess multicompartment body and shell compositions with specifically designed geometries, as shown by the images in Figure 21f.^206^ These microfibers are employed for cell encapsulation and culture matrices, and the results indicate that the all‐aqueous jet‐based microfibers enable confined growth of encapsulated cells along their internals, which would be useful tools for cell patterning.^207^

## 7 Applications: Toward Functional Artificial LLPS

All‐aqueous microfluidics create an excellent eco‐friendly environment for fabricating intracellular‐inspired biomaterials with elaborately designed structures, high biocompatibility, and well‐tailored functionalities, which can be harnessed in many applications, including the production of cosmetics and medicine, food industry, tissue engineering, sorting and extraction of cells and biomolecules, chemical/biochemical analysis, and so on.^185^, ^208^, ^209^, ^210^, ^211^, ^212^, ^213^ Here, we focus the discussion on emerging biomedical applications including drug delivery vehicles and bioreactors, artificial liquid organelles, as well as patterning of cells and culture of organoids.

### 7.1 Drug Delivery Vehicles and Bioreactors

**Figure 22:**
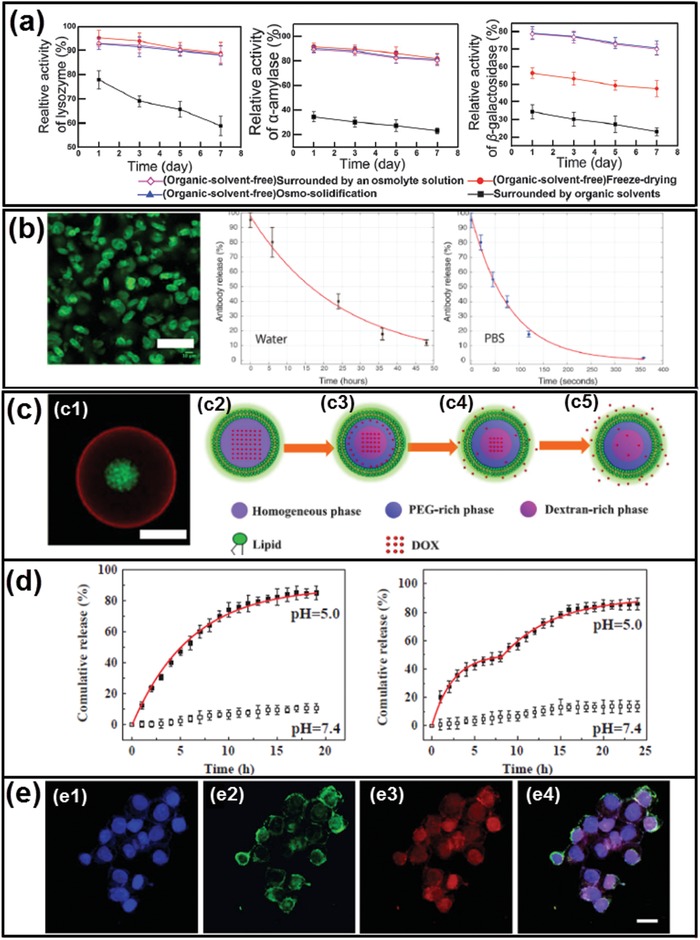
Biomaterials fabricated from all‐aqueous microfluidics for encapsulating and delivering active proteins and therapeutic drugs. a) Relative activity of lysozyme, ɑ‐amylase, and β‐galactosidase dissolved in the same droplet phase after exposure to an organic solvent and an aqueous osmolyte solution, subjected to freeze‐drying and osmo‐solidification for 7 days. b) Antibody release from alginate‐based hydrogel particles; scale bar: 50 µm. c) Schematic illustration of preparation and stimulation of ATPS‐liposomes for encapsulating and releasing DOX. d) DOX release profiles of (left) simple aqueous liposome and (right) ATPS‐liposome. e) Fluorescence microscope images of the uptake of NBD‐PE labeled DOX loaded ATPS‐liposomes by HeLa cells; scale bars: 20 µm. a) Reproduced with permission.^76^ Copyright 2016, Royal Society of Chemistry. b) Reproduced with permission.^215^ Copyright 2015, Wiley‐VCH. c–e) Reproduced with permission.^113^ Copyright 2018, Elsevier.

One of the most intriguing applications of all‐aqueous microfluidic emulsions templated intracellular‐inspired biomaterials is their use as novel delivery systems for labile biomolecules.^169^, ^173^, ^214^ Since there are no harmful organic solvents involved, the activity of the bioactive ingredients could be well protected when encapsulated in the fabricated biomaterials. For instance, when encapsulated in the osmo‐solidified microparticles,^76^ the activities of enzymes, such as lysozyme, β‐galactosidase, and ɑ‐amylase, can be well preserved for 7 days with the remaining activity higher than 90%. Further, compared with other conventional solidification approaches, such as freeze drying, enzyme activities can be enhanced to an unprecedentedly high level when encapsulated in osmo‐solidified microparticles, as demonstrated by the plots in **Figure 22**a.^76^ Enzymes can be used as sensors as long as they retain their activity. Therefore, chemical and physical stability of enzymes are essential requirements in forming enzymatic biosensors. Besides, hydrogel particles generated from all‐aqueous microfluidic emulsions have also been used as superior vehicles for encapsulating and releasing antibodies, as shown in Figure 22b.^215^ Specifically, FITC‐labeled mouse IgG can be successfully encapsulated in the alginate biconcave particles similar the dimensions of mammalian cells, as shown in the fluorescent image in Figure 22b. Afterward, the release kinetics of IgG from the alginate particles is systematically investigated. The alginate particles are dissolved in phosphate buffered saline (1× PBS) or pure water, and the release of IgG is monitored over time. The results show that the release of IgG in water is extremely slower than that in PBS solution, possibly due to the high free calcium concentrations in PBS solution, which will result in loosening the matrix of the particles,^216^ as demonstrated by the plots in Figure 22b. Therefore, due to slow degradation of alginate particles in aqueous fluids, the release of therapeutic compounds should be extended, offering an approach to improve the biological action of therapeutics.

Besides active proteins, therapeutic drugs can also be delivered in the fabricated biomaterials. For example, doxorubicin (DOX) can be loaded in the pH‐sensitive liposome‐coated ATPS droplets, as shown by the CLSM image in Figure 22c.^113^ Initially, the ATPS droplets are homogeneous above phase transition temperature; when cooling down to room temperature, it will separate into PEG‐rich phase and dextran‐rich phase, respectively. Upon the change of pH and temperature, DOX can be distributed in the two phases disproportionally and the overall release of DOX can be divided into two stages and prolonged when compared with simple aqueous liposomes, as illustrated schematically in Figure 22c and demonstrated by the plots in Figure 22d. Moreover, the uptake efficiency of DOX‐loaded liposome‐coated ATPS droplets by HeLa cells is 2.75‐folds higher than that of free DOX, indicating that the fabricated biomaterials have great potential in improving the pharmacologic therapy of cancer treatment, as demonstrated by the fluorescence images in Figure 22e.

**Figure 23:**
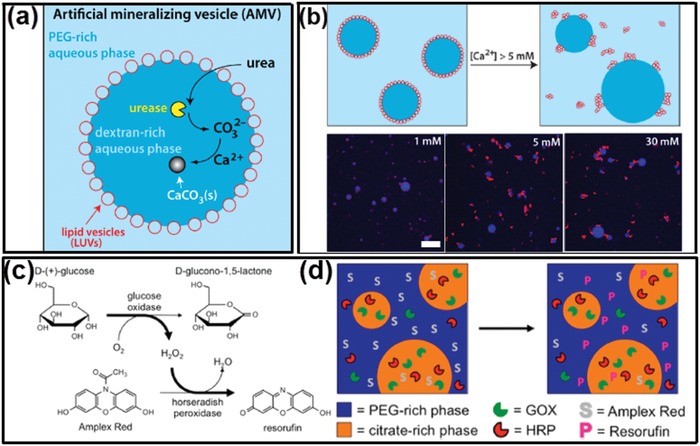
Biomaterials fabricated from all‐aqueous microfluidics as biomimetic microreactors. a) Schematics of enzymatic production of CaCO_3_ mineral within liposome‐coated ATPS vesicles. b) (Upper line) Schematics of the experiment, in which the effect of adding Ca^2+^ to a sample of liposome‐coated ATPS vesicles is evaluated. At 5 × 10^−3^ m Ca^2+^ and above, the interfacial layer of liposomes is disrupted. (Bottom line) CLSM images of liposome‐coated ATPS vesicles with different amounts of added Ca^2+^ as indicated in each panel. Scale bar: 10 µm. c) The sequential enzyme reaction of GOX and HRP with the corresponding substrates and products. d) Schematic of the GOX and HRP catalyzation in an ATPS‐based heterogeneous media. a,b) Reproduced with permission.^211^ Copyright 2015, American Chemical Society. c,d) Reproduced with permission.^220^ Copyright 2014, American Chemical Society.

Apart from drug delivery vehicles, all‐aqueous microfluidic templated vesicles and capsules are useful bioreactors since they can maintain reaction‐relevant internal environments, while allowing the entry and exit of substrates and products in mild conditions.^95^ Previously, we have discussed the fabrication of liposome‐coated vesicles by incorporating liposomes at all‐aqueous interfaces,^154^, ^211^ these liposome‐coated vesicles can be applied as bioreactors for mimicking intracellular mineral deposition within living cells. The liposome‐stabilized all‐aqueous interfaces allow the entry of precursors while retaining an enzyme catalyst by equilibrium partitioning between internal and external aqueous phases. Small molecule chelators are employed to control Ca^2+^ availability during CaCO_3_ mineralization, providing protection against liposome aggregation while generating CaCO_3_. Mineral deposition is limited in the interior due to the localized production of CO_3_^2−^ by compartmentalized urease, as illustrated schematically in **Figure 23**a,b. This approach can be adaptable for enzyme‐catalyzed synthesis of a wide variety of materials, by varying the type of metal ion and enzyme. Moreover, Benefiting from the preferential partitioning of the all‐aqueous emulsions, the templated architectures can be applied as bioreactors for mimicking intracellular biological reactions within living cells.^172^, ^217^, ^218^, ^219^ For instance, these bioreactors can be utilized to perform enzymatic reactions between the glucose oxidase (GOX) and horseradish peroxidase (HRP) in the PEG/citrate ATPS as shown in Figure 23c,d.^220^ Specifically, the GOX, HRP, and the corresponding substrates glucose and H_2_O_2_ favors the citrate‐rich phase, while the substrate amplex Red partitions to PEG‐rich phase. As a result, the HRP‐catalyzed reaction can only be conducted in the PEG‐rich phase, while the GOX‐catalyzed reaction can be performed in both PEG‐rich and citrate‐rich phase. Such enzymatic reactions with nonequilibrium distributed reactants pave the way of understanding the biological reactions a heterogeneous media such as the cell interiors.

### 7.2 Artificial Membraneless Organelles

**Figure 24:**
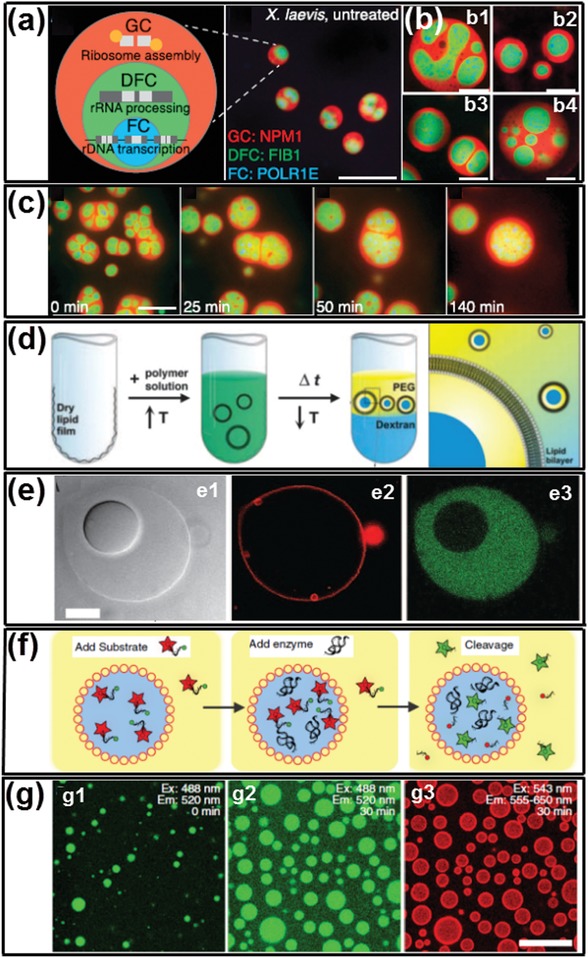
All‐aqueous microfluidics‐templated artificial membraneless organelles. a) Schematics of ribosome biogenesis in nucleolus (right), images showing nucleoli in an untreated *Xenopus laevis* nucleus (left); scale bars: 20 µm. b) Examples of nucleoli after coarsening in *X. laevis* nuclei treated with Lat‐A; scale bars: 20 µm. c) Time‐course of nucleolar component fusion after actin disruption by Lat‐A; scale bar: 20 µm. d) Schematics of liposome‐coated ATPS droplets formation. e1) Transmitted light and e2,e3) fluorescent images of liposome‐coated ATPS droplets, prepared with rhodamine‐tagged lipid (center) and fluorescein‐tagged streptavidin (right); scale bar: 10 µm. f) Schematics of ribozyme cleavage reaction in liposome‐coated ATPS droplets. g) Protein partitioning and RNA cleavage reaction within liposome‐stabilized ATPS. a–c) Reproduced with permission.^118^ Copyright 2016, Elsevier. d,e) Reproduced with permission.^221^ Copyright 2005, National Academy of Sciences. f,g) Reproduced with permission.^172^ Copyright 2014, Springer Nature.

**Figure 25:**
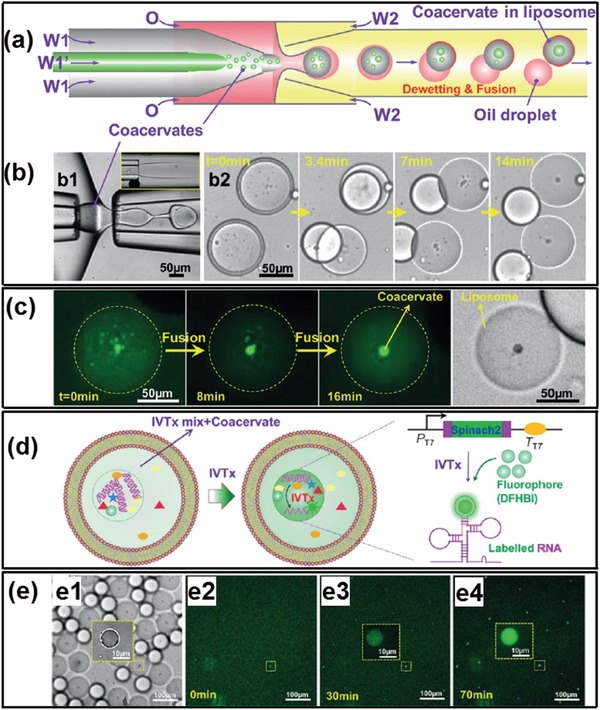
Artificial nucleoid‐like membraneless organelles in liposome‐coated ATPS droplets. a) Schematics and b) optical microscope images of the microfluidic preparation of emulsion templates with coacervates as well as relevant dewetting transition and fusion process to form liposome‐coated ATPS droplets. c) CLSM images of the fusion of small coacervates into a big coacervate to form nucleoid‐like membraneless organelles. d) Illustrations of IVTx in coacervate droplet in liposome and the working principle of detection of generated RNA using aptamer Spinach 2 and dye DFHBI. e1) Optical microscope image of formed liposome‐coated ATPS droplets containing coacervate droplets and IVTx mix; e2–e4) CLSM images show RNA generation in coacervates over time. Reproduced with permission.^222^ Copyright 2017, Wiley‐VCH.

Membraneless organelles, such as subcompartmentalized organization of nuclei illustrated in **Figure 24**a–c,^118^ share a high degree of similarity with all‐aqueous emulsions, both of which are formed due to aqueous–aqueous phase separation in molecular crowded environment and possess droplet‐like properties. The interfacial tensions of both systems are as low as 1 µN m^−1^, and their dynamic interface allows transfer and exchange of biomolecules. Therefore, all‐aqueous emulsion can be viewed as a minimalistic physical model of membraneless organelles. Recent biomimetic studies frequently use all‐aqueous emulsion and (or) liquid coacervates to study and prepare organelle‐like functional droplets. For instance, a single‐phase solution dissolved with dextran, PEG, and lipids will phase separate and assemble into lipid vesicles containing dextran‐rich and PEG‐rich domains, which can subsequently act as a simplified model of membraneless organelles, as illustrated in Figure 24d,e.^221^ Furthermore, RNA can selectively partition into the formed membraneless organelles and ribozyme cleavage reaction can be conducted inside the fabricated liposome‐coated vesicles, as shown in Figure 24f,g.^172^ Moreover, liposome‐coated ATPS droplets have also been used to generate nucleoid‐like membraneless organelles for investigating the in vitro DNA transcription (IVTx). As shown in **Figure 25**a,b, microfluidic channels are used to fabricate liposome‐coated ATPS droplets containing two reactive coacervates, such as RNA and poly‐l‐lysine. As the two coacervates gradually react with each other within the liposome‐coated ATPS droplets, the generated coacervates would fuse into one phase and separate with surrounding aqueous medium, forming membraneless organelles and constructing the nucleoid‐like structure, as demonstrated by the CLSM images in Figure 25c.^222^ In addition, IVTx can be performed in the generated artificial membraneless organelles. As shown in Figure 25d,e, the complex coacervates composed of spermidine and polyuridylic acid (polyU) together with IVTx components are encapsulated in the liposome‐coated ATPS droplets. The sequence of CLSM images in Figure 25e shows high fluorescence in the nucleoid‐like membraneless organelles due to RNA synthesis, while a lower signal was observed in the surrounding water shells, demonstrating that transcription exclusively occurs in the fabricated nucleoid‐like membraneless organelles.

### 7.3 Cell Patterning and Microtissue Culturing

The soft and dynamic interfaces of all‐aqueous emulsions, along with their inherent preferential partitioning properties, allow for the active ingredients and living cells to partition and compartmentalization in emulsions and in the generated biomaterials, which further creates possibilities of novel 3D cell spheroid formation^223^ for high‐throughput drug screening tests and microtissue culturing.

**Figure 26:**
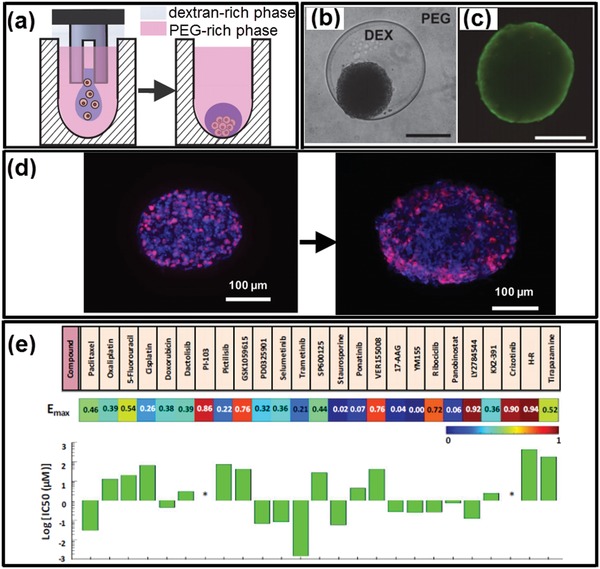
All‐aqueous microfluidics‐templated 3D cancer cell spheroids formation for high‐throughput drug screening tests. a) Dispensing a dextran‐rich phase drop containing cells into a microwell containing the PEG‐rich phase to result in the formation of cell spheroid within the drop during incubation. b,c) Optical and fluorescent microscope images of resultant spheroids of MDA‐MB‐157 breast cancer cells. Scale bars: 250 µm. d) Proliferation of formed cell spheroids during incubation. e) Demonstration of the anticancer compounds screening by using formed HT‐29 cell spheroids. a–c) Reproduced with permission.^227^ Copyright 2015, Springer Nature. d) Reproduced with permission.^228^ Copyright 2016, Wiley‐VCH. e) Reproduced with permission.^226^ Copyright 2016, American Chemical Society.

ATPS can be used as the physiological template to facilitate the formation of 3D tumor cell spheroids which can be applied for screening candidate oncologic compounds and drugs.^224^, ^225^ By taking advantage of their preferential partitioning in different phases of ATPS, various cancer cells, such as HT‐29 colon cancer cells, U‐87 MG brain cancer cells, and MDA‐MB‐157 breast cancer cells, have been patterned in microwells to form 3D tumor spheroids^226^ for high‐throughput anticancer drug screening, as illustrated schematically in **Figure 26**a. The cultivated spheroids show spherical morphology in high reproducibility (Figure 26b) and excellent viability (Figure 26c) to proliferate into larger spheroids (Figure 26d).^226^, ^227^, ^228^ The patterned tumor spheroids partially addressed challenges in the heterogeneity of spheroid culture in vitro, which is therefore used as promising platforms for executing high‐throughput anticancer compounds screening. The ability to scale‐up 3D spheroid culture in microfluidic devices offers new opportunity for fast testing of multiple anticancer drugs and analyze their half‐maximum (IC_50_) and maximum (*E*_max_) inhibitory concentrations, which facilitates the multiparametric analysis of cellular responses to drug compounds and identify novel chemical probes with chemotherapeutic benefits,^226^ as demonstrated in Figure 26e.^229^

**Figure 27:**
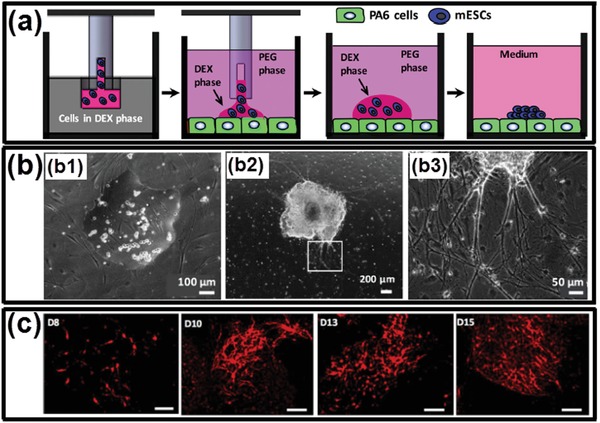
All‐aqueous microfluidics‐templated stem cell patterning and microtissue culturing. a) Schematics illustrating the formation process of the stem cell colony. b) Optical microscope images of embryonic stem cells within a dextran drop on PA6 cells immersed in the aqueous PEG phase. b1,b2) Embryonic stem cells proliferate and form a single colony; the image shows a colony on day 8 of co‐culture. b3) Magnified view from panel (b2) shows that neurite processes emerge from differentiating embryonic stem cells in the colony; the underlying PA6 cells remain completely intact during the two‐week co‐culture. c) Immunocytochemical analyses of TH stained images of dopaminergic neurons during the second week of co‐culture of embryonic stem cells and PA6 cells. Scale bars: 100 µm. a,b) Reproduced with permission.^233^ Copyright 2017, Oxford University Press. c) Reproduced with permission.^45^ Copyright 2016, Public Library of Science.

Moreover, in the area of developmental biology, all‐aqueous microfluidics have shown to control the localization of stem cells over a layer of supporting stromal cells to cultivate microtissues.^230^, ^231^, ^232^ Upon receiving physical and chemical cues from the stromal cells, the patterned stem cells can differentiate toward specific tissues. For instance, ATPS‐based heterocellular culture systems that resemble embryonic development in terms of direct intercellular interactions and induce neural differentiation of embryonic stem cells have been developed. Embryonic stem cells are dispersed in dextran/PEG ATPS and patterned on the surface of PA6 stromal cells to form a defined stem cell niche,^233^ as illustrated in **Figure 27**a. When subjected to various stimuli from the PA6 cells, embryonic stem cells can differentiate toward specific neural lineages such as neurons, astrocytes, and oligodendrocytes, subsequently leading to the generation of neural microtissues, as shown in Figure 27b. The formed neural microtissues can be used as novel tools for investigating the cellular and molecular modulation of the biological differentiation process,^45^ as demonstrated in Figure 27c.

## 8 Conclusions and Outlooks

In summary, we have outlined the recent progress in the development of cell‐inspired all‐aqueous microfluidics by overviewing the features of intracellular liquid–liquid phase separation. The principles underlie the fluid physics, microfluidic techniques to manipulate all‐aqueous structures, approaches to fabricate biomaterials, and their emerging biomedical applications are summarized. The robustness to generate complicated all‐aqueous structures, the superior biocompatibility to generate biomaterials, as well as the upscaled efficiency and reproducibility in bioengineered systems from intracellular LLPS, making them exquisite templates for fabrication of advanced materials and systems. The cell‐inspired concepts and the “green” microfluidic technology will lead to better understanding in fundamental biological science and propel new advancements in biomedical techniques and applications. A plenty of rising opportunities, together with challenges, are briefed below.

### 8.1 Cytomimetic All‐Aqueous Emulsions

Most of the current studied all‐aqueous emulsions use salts and polymers as hydrophilic additives to form aqueous solutions, such as the dextran/PEG system and the PEG/salt system. However, to better mimic the intracellular LLPS and facilitate deeper understanding of cytophysics and cytopathology, finding new cytomimetic all‐aqueous emulsions becomes the major research trend in the last decade.^25^, ^135^, ^172^, ^221^ Recently, coacervates formed by intrinsically disordered proteins (IDPs) and IDP‐binding RNA have been employed as novel ATPS systems. The immiscibility of their biomolecules is encoded by their primary (sequence‐dependent) and secondary structures.^234^, ^235^ These organelle‐like ATPSs have shown responsiveness to environmental stimulus, such as temperature, pH, and different biological signals. For instance, IDPs with different amino acid sequences and compositions exhibit tunable lower or upper critical solution temperature (LCST and UCST, respectively) transitions in physiological conditions. Furthermore, the secondary structure of mRNA defines the ability of RNA to engage in homo‐ or heteromeric interactions and thus drives specificity in the resultant RNA–RNA coacervate‐induced phase separation. These “liquid coacervates” are formed by the multivalent weak interactions between two or more macromolecules^236^, ^237^ and are a novel type of all‐aqueous emulsions. Inspired from this system, new areas to study phase transition behavior of membraneless organelles and their correlations with organelle diseases have been boosted, and many interdisciplinary researches in materials science, physic chemistry, biology as well as biotechnology, and medicine are growing.^238^, ^239^, ^240^

### 8.2 Cell‐Inspired Exquisite Strategies to Manipulate All‐Aqueous Emulsions

All‐aqueous emulsions have ultralow interfacial tension and high dynamic viscosity; therefore, external inputs, such as hydrodynamic perturbations and electrospray, are often adopted to achieve the generation of all‐aqueous droplets. However, the dimension and the generation rate of the all‐aqueous droplets are limited by the current strategies, typically with diameters no less than 10 µm and with generation frequency of less than 10^2^ Hz.^77^ While the liquid coacervates in biological systems always have much smaller sizes with diameters below 10 µm,^241^, ^242^ the current all‐aqueous microfluidic technique has not develop enough proficiency in controlling the size and sophistication exhibited by their biological counterparts. Therefore, to further reduce the dimension of the all‐aqueous droplets to sub‐micrometer size range and increase the generation frequency to orders of magnitude, new strategies have been proposed and investigated, such as nanofluidics^243^, ^244^ and electric‐driven or light‐driven microfluidics.^245^, ^246^, ^247^ Moreover, the investigation of all‐aqueous emulsions in sub‐micrometer and nanometer size will bring new opportunities for fundamental studies at cellular or even subcellular level, such as the development of the protein/RNA‐based biological liquid coacervates for investigating organelle physics and physiology.^248^, ^249^, ^250^

### 8.3 Intracellular Inspirations for Advanced Material Construction and Applications

While numerous materials have been inspired from cells and subcellular structures, most of the concepts are still at their infancy and worthy of further development. Recent studies on the temperature‐dependent phase transition behaviors of IDPs in membraneless organelles have revealed a strong relation with the composition and sequence of their amino acids. Interestingly, an increase in the number of repetitive segments in IDPs leads to a higher propensity of droplet formation.^234^ Composition of the repetitive amino acid sequence often defines the critical temperature of phase transition (either UCST or LCST).^234^, ^235^ With the understanding, engineered elastin‐like peptides (ELPs) have been designed with liquid–liquid phase transition behaviors occurring around the physiological temperature (≈37 °C).^251^ When ELPs are crosslinked or hybridized with graphene, the resulting hydrogels can be made into shape‐memory hydrogels, thermosensors, light‐controlled actuators, and other flexible electronics.^252^, ^253^ Moreover, the phase transition behaviors of IDPs, together with their catalytic function, are often activated upon combining with IDP‐binding RNA during the formation of liquid organelles, which inspires novel design of electric valves in semiconductors, biosensors, robots, and other smart devices.^254^, ^255^ Furthermore, understanding of the reversibility of the liquid–liquid phase transition of membraneless organelles offers new promises to the diagnosis and therapeutic control of organelle diseases. In diseased membraneless organelles, an abnormal coacervation between RNA and RNA‐binding proteins may lead to irreversible liquid–solid transition that frequently correlates with neurodegenerative diseases and dyskinesia. Examples include amyotrophic lateral sclerosis induced by the fibration of FUS proteins,^256^ Alzheimer's Disease induced by the abnormal deposition of tau protein,^257^, ^258^ and Ewing's sarcoma induced by aberrant accumulation of proteins near the genome associated with tumorigenesis.^259^ The biomimetic construction of liquid organelle‐like materials in vitro provides a simplified alternative to understand the pathological phase transition of diseased organelles, thus shedding lights on the understanding of mechanism of neurodegenerative diseases and cancers. Besides, microfluidic preparation of organelle‐like materials also provides a platform to the preliminary screening and rapid testing of small molecular drugs targeting for specific organelle diseases.^260^, ^261^, ^262^ The membraneless organelles inspired biomaterials therefore are of great clinical importance in both disease study and treatment.

## Conflict of Interest

The authors declare no conflict of interest.
